# Genome-Wide Distribution, Organisation and Functional Characterization of Disease Resistance and Defence Response Genes across Rice Species

**DOI:** 10.1371/journal.pone.0125964

**Published:** 2015-04-22

**Authors:** Sangeeta Singh, Suresh Chand, N. K. Singh, Tilak Raj Sharma

**Affiliations:** 1 National Research Center on Plant Biotechnology, Pusa Campus, New Delhi, 110012, India; 2 School of Life Sciences, Devi Ahilya University, Khandwa Road, Indore, 452017, India; UCLA-DOE Institute for Genomics and Proteomics, UNITED STATES

## Abstract

The resistance (R) genes and defense response (DR) genes have become very important resources for the development of disease resistant cultivars. In the present investigation, genome-wide identification, expression, phylogenetic and synteny analysis was done for R and DR-genes across three species of rice viz: *Oryza sativa ssp indica cv 93-11*, *Oryza sativa ssp japonica* and wild rice species, *Oryza brachyantha*. We used the *in silico* approach to identify and map 786 R -genes and 167 DR-genes, 672 R-genes and 142 DR-genes, 251 R-genes and 86 DR-genes in the *japonica*, *indica* and *O*. *brachyanth* a genomes, respectively. Our analysis showed that 60.5% and 55.6% of the R-genes are tandemly repeated within clusters and distributed over all the rice chromosomes in *indica* and *japonica* genomes, respectively. The phylogenetic analysis along with motif distribution shows high degree of conservation of R- and DR-genes in clusters. *In silico* expression analysis of R-genes and DR-genes showed more than 85% were expressed genes showing corresponding EST matches in the databases. This study gave special emphasis on mechanisms of gene evolution and duplication for R and DR genes across species. Analysis of paralogs across rice species indicated 17% and 4.38% R-genes, 29% and 11.63% DR-genes duplication in *indica* and *Oryza brachyantha*, as compared to 20% and 26% duplication of R-genes and DR-genes in *japonica* respectively. We found that during the course of duplication only 9.5% of R- and DR-genes changed their function and rest of the genes have maintained their identity. Syntenic relationship across three genomes inferred that more orthology is shared between *indica* and *japonica* genomes as compared to *brachyantha* genome. Genome wide identification of R-genes and DR-genes in the rice genome will help in allele mining and functional validation of these genes, and to understand molecular mechanism of disease resistance and their evolution in rice and related species.

## Introduction

Rice (Oryza sativa*)* is one of the most important food crops of the world and its yield is constantly affected by several diseases [1]. More than 70% diseases caused by fungi, bacteria, viruses and nematodes have been recorded on rice, among these rice blast (*Magnaporthe oryzae*), bacterial leaf blight (*Xanthomonas oryzae pv*.*oryzae*) and sheath blight (*Rhizoctonia solani*) are the most serious constraints affecting rice productivity [2]. The severity and significance of damage caused by pathogens in rice have necessitated the development of effective disease management strategies to minimize the crop losses. Among such new strategies, the exploitation of host resistance appears to be the most reliable method of disease management.

Disease resistance mechanism can be better understood by the identification of host genes involved in defense response. The resistance to a particular pathogen only occurs when the pathogen carries a specific avirulence (Avr) gene and the plant carries a corresponding R-gene [3]. Plants will be resistant to the pathogens when compatible R- and Avr-genes are present in host-pathogen systems [4]. This gene-for-gene interaction is very specific and important in getting resistance phenotypes [5]. Resistance genes belong to a very large multigene family, have diverse recognition specificities and are highly polymorphic [6]. Genes conferring resistance to the major classes of plant pathogens have been isolated and well characterized from different plant species [7]. Analysis of predicted proteins of R-genes revealed presence of common motifs in the cloned resistance genes of diverse origin and pathogen specificity [8, 9]. The clustered distribution of R-genes provides a reservoir of genetic variation from which new specificities can evolve. Various molecular mechanism like gene duplication, unequal crossing over, ectopic recombination, and diversifying selection have been proposed to contribute to the structure of R-gene clusters and the evolution of resistance specificities [10].

To date, over 100-R genes have been cloned (www.prgdb.org) and some of them are well characterized [11]. Based on their structural similarity, the cloned R-genes can be grouped into 5 classes [12] such as i. cytoplasmic receptor-like protein with nucleotide binding site (NBS) and a leucine rich repeat (LRR) domain, ii. A serine- threonine kinase, iii. Trans membrane receptors with a large extra cytoplasmic LRR domain, iv. Transmembrane receptors with a large extracellular serine-threonine kinase domain and, v. Receptor with HC-toxin reductase. The predicted R-proteins contain several common structural motifs like NBS, LRR, transmembrane domains (TM), and serine/threonine protein kinases (PK). The NBS-containing proteins are necessary for many fundamental eukaryotic cellular events such as cell growth, differentiation, cytoskeletal organization, vesicle transport, and defense [13]. Therefore, NBS domains have been the subject of structure-function analysis in other proteins. The LRRs have been demonstrated in protein-protein interactions and ligand binding in signal-transducing eukaryotic proteins [14]. The LRR has functional importance in disease resistance response, because single amino acid changes in the LRR domain of R-genes such as *RPS2*, *RPM1*, *RPS5*, and *N* affect resistance phenotype [15, 16]. These results suggest that the function of the LRR domain can be eliminated by minor modifications.

The NBS encoding genes belong to one of the largest gene families in plant genomes, and have been identified in all plant species [17]. All angiosperms evaluated to date contain NBS-LRR encoding genes, but differences exist between monocot and dicot species. While more than half of the NBS-encoding genes identified in *Arabidopsis thaliana* code for TIR domains [18], members of this subclass appear to be absent in cereal species [19, 20]. This finding suggests that since divergence (~200 million years ago), association of TIR domain with NBS-encoding genes was preserved by dicots but lost in monocots [21]. The NBS resistance gene families have been evaluated in numerous plants including *Arabidopsis thaliana* [18, 22], *Populus trichocarpa* [23, 24], *Rosa roxburghii* “Chestnut Rose” [25], *Saccharum spp*. [26], *Ipomoea batatas Lam*. [27], *Medicago truncatula* [28], *Oryza sativa* [29, 19, 20], *Vicia faba L*. [30], *Cicer arietinum L*. [30], *Prunus armeniaca L*. [31], *Helianthus annuus L*. [32], *Medicago sativa L*. [33], *Vitis vinifera* [24] and *Cajnus cajan* [34]. In these studies, it has been reported that the ancient NBS-LRR super family represents the largest class of disease resistance genes in plants. The orthologs are the genes that diverged as a result of speciation and still have retained identical biological function. Higher number of orthology for a gene family between species may reflect high conservation of their function in those species [35].

With the availability of complete rice genome sequence it has become easier to study about disease resistance and defense response genes. Sequencing of cultivated rice, *O*. *japonica*, *O*.*indica* and wild rice *O*.*brachyanta* [36] have made the comparative analysis with respect to R- and DR-genes easier. *O*.*brachyantha* is a distant relative of cultivated rice (*O*. *sativa Japonica* and *O*. *sativa Indica*). Despite the importance of such genes in crop improvement breeding programmes, no automatic annotation tools are yet available. This may be explained by the complex nature of R-genes belonging to different classes and also have limited number of related functional domains [37]. Thus it is important to understand the nature of these genes in order to overcome the difficulties involved in their automatic annotation [38]. Earlier studies on *in silico* analysis of rice genome for resistance genes were focused on *indica* and *japonica* genomes but detailed analysis of R- and DR-genes for wild rice species was lacking. In view of the importance of disease resistance and defense response genes in the resistance phenotypes these need to be studied in detail at the whole genome level. Earlier studies on rice were focused on NBS-LRR category of the genes [20], but a critical analysis of all types of resistance- and defense response- genes, their physical position and orientation on the chromosomes, clustering behavior, evolution and extent of their expression levels have not been studied well. Because defense response genes like glucanases, chitinases and thaumatin-like protein play a vital role in defense mechanism, they need to be studied in detail, which have been not done earlier. Mapping of R-genes and DR-genes will provide insights to the genome organization of functionally related genes. In view of foregoing the objectives of present investigations were (i) identification of different categories of R- and DR- genes in highly curated rice genome sequence data, (ii) defining exact physical position of each genes on different chromosomes (iii) understanding clustering nature, evolution and organization of these genes (iv) *in silico* gene expression analysis, (v) study of gene duplication through paralogy analysis and (vi) to understand orthologous relation between different rice genomes.

## Results and Discussion

### Distribution of resistance genes across rice species

**Fig 1:**
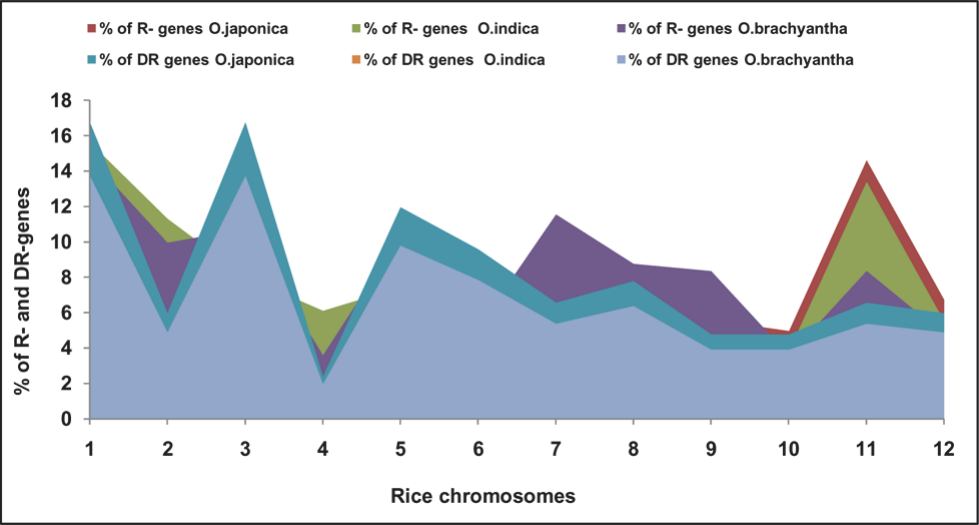
Distribution of R- and DR-genes. Percentage distribution of R- and DR-genes on each chromosome of O.*sativa ssp japonica*, *O*. *sativa ssp indica* and *O*. *brachyantha* species.

**Fig 2:**
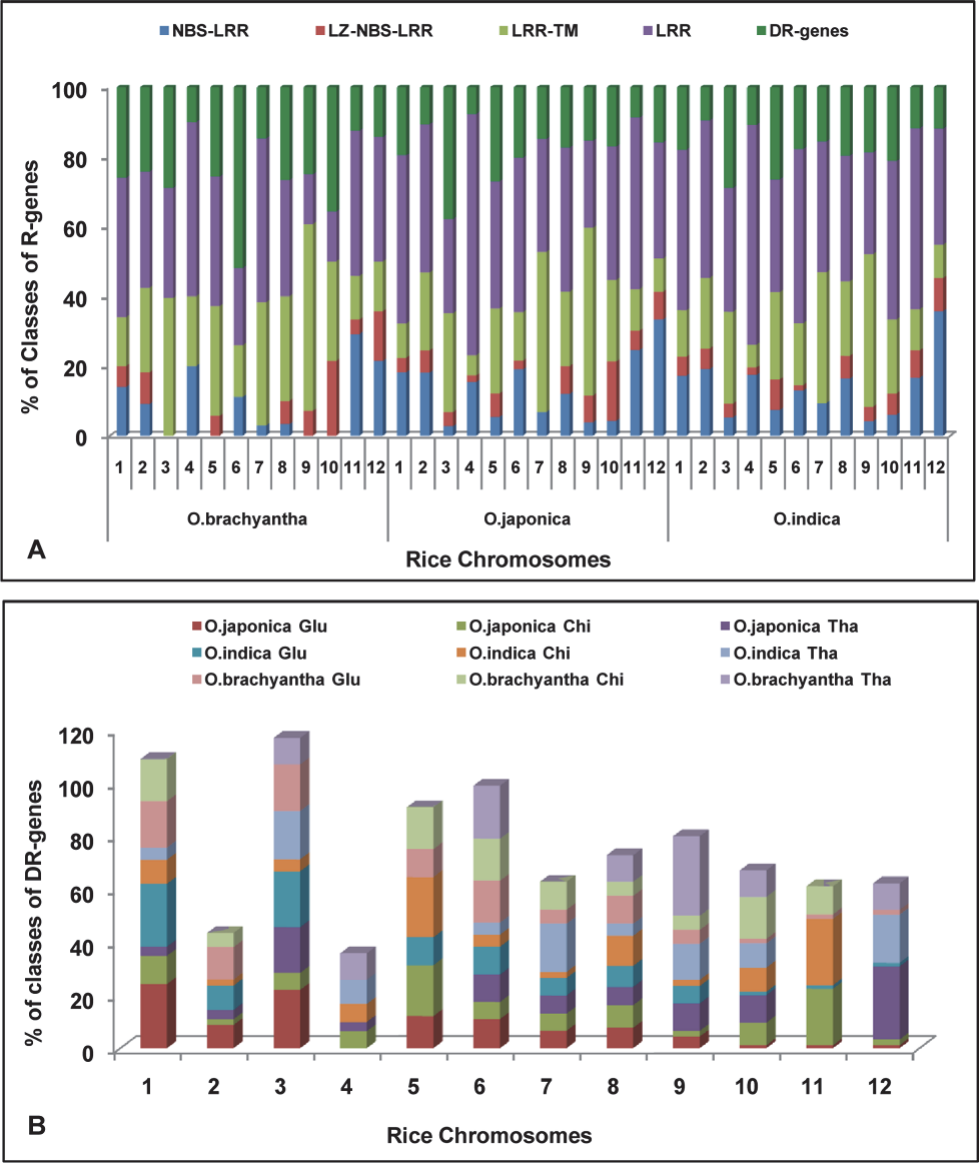
Percentage distribution of classes of R- and DR-genes. (A) Percentage distribution of R-genes belonging to specific class present on 12 chromosomes of *O*. *sativa ssp japonica*, *O*. *sativa ssp indica* and *O*. *brachyantha* species. (B) Percentage distribution of DR-genes belonging to specific class present on 12 chromosomes of *O*. *sativa ssp japonica*, *O*. *sativa ssp indica* and *O*. *brachyantha* species.

We used a data set of 61250 rice cDNA and identified 786-R gene models in rice genome of *japonica* species, which is 1.16% of the total number of the gene models predicted in rice (S1 Table). There was uneven distribution of R-genes on the chromosomes with a higher frequency of occurrence at some loci (Fig 1). Maximum numbers (115) of R-genes were identified on chromosome 1 and chromosome 11. Least number (39) of R-genes was identified on chromosome 10. R-genes in both long and short arm of chromosome 1 were equally distributed. Whereas the number of genes on long arm of chromosome 11 are just double the number present on short arm. Out of 786 predicted R-genes, 50% (396) belongs to LRR category, which includes the genes containing LRR motif but without NBS, CC or LZ motifs (Fig 2A). Of the total R-genes predicted in rice genome, 24% of the genes have homology to LRR-TM category. The extensively studied *Xa21* gene belongs to LRR-TM category, mapped and cloned from chromosome 11 also has many copies on chromosomes 2, 8 and 11. Similarly 142 genes showed homology to the NBS-LRR class of R-like genes and 53 genes have homology to LZ-NBS-LRR class of R-like genes. Under various keywords used to search NBS-LRR class (eg. *Pib*, *Pita*, *Rp 1-d8*, *LR10*, *Mla 1*, rust resistance and NBS-LRR), the orthologues of *Rp1* were found on chromosomes 1, 2, 4 and 12 and stem rust resistance like proteins found at six positions on chromosome 11. The orthologues of *Mlo* and *Pib* like genes were found to be located on chromosome 12. We have also analyzed orientation of all the genes present in rice genome which will help in their eventual cloning and characterization.

In *O*.*sativa ssp indica* we predicted 52080 *indica* rice genes and used them as data set to identify 672-R gene models in rice genome (S1 Table). There was uneven distribution of R-genes on the chromosome with a higher frequency of occurrence at some loci (Fig 1). Maximum numbers (105) of R-genes were identified on chromosome 1 followed by chromosome 11. Least number (26) of R-genes was identified on chromosome 10. Out of 672 predicted R-genes, 51% (348) belongs to LRR category, which includes the genes containing LRR motif but without NBS, CC or LZ motifs (Fig 2A). Of the total R-genes predicted in rice genome, 24% of the genes have homology to LRR-TM category. Similarly 115 genes showed homology to the NBS-LRR class of R-like genes and 43 genes have homology to LZ-NBS-LRR class of R-like genes.

In the wild species of rice *Oryza brachyantha*, we predicted 28873 gene models and used them as data set to identify 251-R gene models in its genome (S1 Table). Maximum numbers (37) of R-genes were identified on chromosome 1 followed by chromosome 7. Least number (9) of R-genes was identified on chromosome 10 (Fig 1). Out of 251 predicted R-genes, 45% (114) belongs to LRR category. Of the total R-genes predicted in rice genome, 36% of the genes have homology to LRR-TM category. Similarly 27 genes showed homology to the NBS-LRR class of R-like genes and 18 genes have homology to LZ-NBS-LRR class of R-like genes (Fig 2A).

Abundance and diversity of resistance genes have been studied in sugarcane [38], Arabidopsis [18, 22], eucalyptus [39], chickpea [30] and populous [23] etc. Earlier works on rice were focused on NBS-LRR category of R-genes [20] but the detailed genome wide study for all classes of R- and DR-genes with respect to their expression analysis, exact physical position on chromosomes and orientation on each strand of chromosomes has not been studied. Also previous studies on R-genes of rice chromosomes have been done on 11^th^ and 12^th^ chromosomes [40] while this study is extension of previous works for the complete rice genome with latest version of TIGR release 6.1.In this analysis, the chromosomes 1 and 11 are rich in R-genes. Earlier it has also been reported that most of the R-like genes (24.98%) present on chromosome 11 [20]. The identification of large number of NBS genes were variable even in earlier predictions [9] and it was reported that rice carries many more of these sequences than Arabidopsis [18]. The annotation of the *Arabidopsis thaliana* genomic sequence [41] recognizes 207 genes with coding domain characteristic of plant resistance proteins, of which 149 belong to the largest class of NBS-LRR [18]. It has been found that the ancient NBS-LRR super family represents the largest class of plant resistance genes. In cereal genomes, it is estimated that ~1% of all genes encodes NBS domains [19]. Some dicot species also contains large numbers of NBS-LRR genes as well. The *Medicago truncatula* genome is estimated to contain approximately 400–500 NBS-LRR genes [28], and in sunflower, 630 NBS-LRR homologs were identified [32]. However in papaya, only ~0.2% encode NBS domains out of a total of 24,746 predicted genes [42]. Whereas in other sequenced genomes like *P*. *trichocarpa*, *A*. *thaliana*, and *V*. *vinifera*, 416 (0.91%), 174 (0.68%), and 535 (1.76%) genes, respectively have been reported as resistance like genes [24]. Therefore, identification of genes responsible for disease resistance in the highly curated version (ver. 6.1) of rice genome would be more accurate compared to the earlier reports. A large number of genes in present study formed LRR category, which includes LRR motif and also genes not included in other four classes. Large number of LRR found in this genome wide study of R-genes is of great significance because LRR has functional significance in disease resistance response and single amino acid changes in the LRR domain eliminates its function of resistance because of lack of recognition specificity between host and pathogen proteins.

### Distribution of defense response genes in the rice genome

The whole genome sequence of *japonica* rice was also analyzed for the identification of defense response (DR) genes. A total of 167 DR-genes were identified and categorized in three classes such as chitinases, glucanases and thaumatin like proteins (Fig 2B). Of these, 47 were identified as chitinases, 29 as thaumatin-like proteins and 91 as glucanases type of genes (S1 Table). Distribution of DR genes was not uniform and in many cases these were found in clusters with tandemly repeated arrangement. Maximum number of defense response genes (28) were found on chromosome1 and 3. DR-genes have been studied for the whole genome which was lacking in previous works [20].

The whole genome sequence of *indica* rice was also analyzed for the identification of defense response (DR) genes (Fig 2B). A total of 142 DR-genes were identified and categorized in three classes such as chitinases, glucanases and thaumatin like proteins. Of these, 44 were identified as chitinases, 22 as thaumatin-like proteins and 76 as glucanases type of genes (S1 Table). Distribution of DR genes was not uniform and in many cases these were found in clusters with tandemly repeated arrangement

The whole genome sequence of *O*.*brachyantha* was also analyzed for the identification of defense response (DR) genes (Fig 2B). A total of 86 DR-genes were identified and categorized in three classes such as chitinases, glucanases and thaumatin like proteins. Of these, 19 were identified as chitinases, 10 as thaumatin-like proteins and 57 as glucanases type of genes (S1 Table). Maximum number of defense response genes (14) were found on chromosome 6 followed by chromosome1 (13).

This analysis of identifying number and orientation of different categories of DR-genes across three genomes of rice has been done for the first time. Earlier works on DR genes were focused on 11^th^ and 12^th^ chromosome of rice [40] and thus they did not provided sufficient information on DR genes for whole genome of rice. Comparative study of DR-genes across three rice genomes concluded that glucanases was among the three categories of DR–genes found in maximum number in all three genomes. Distribution of DR-genes across twelve chromosomes was not uniform in all three genomes.

### Gene clusters on different rice chromosomes

**Fig 3:**
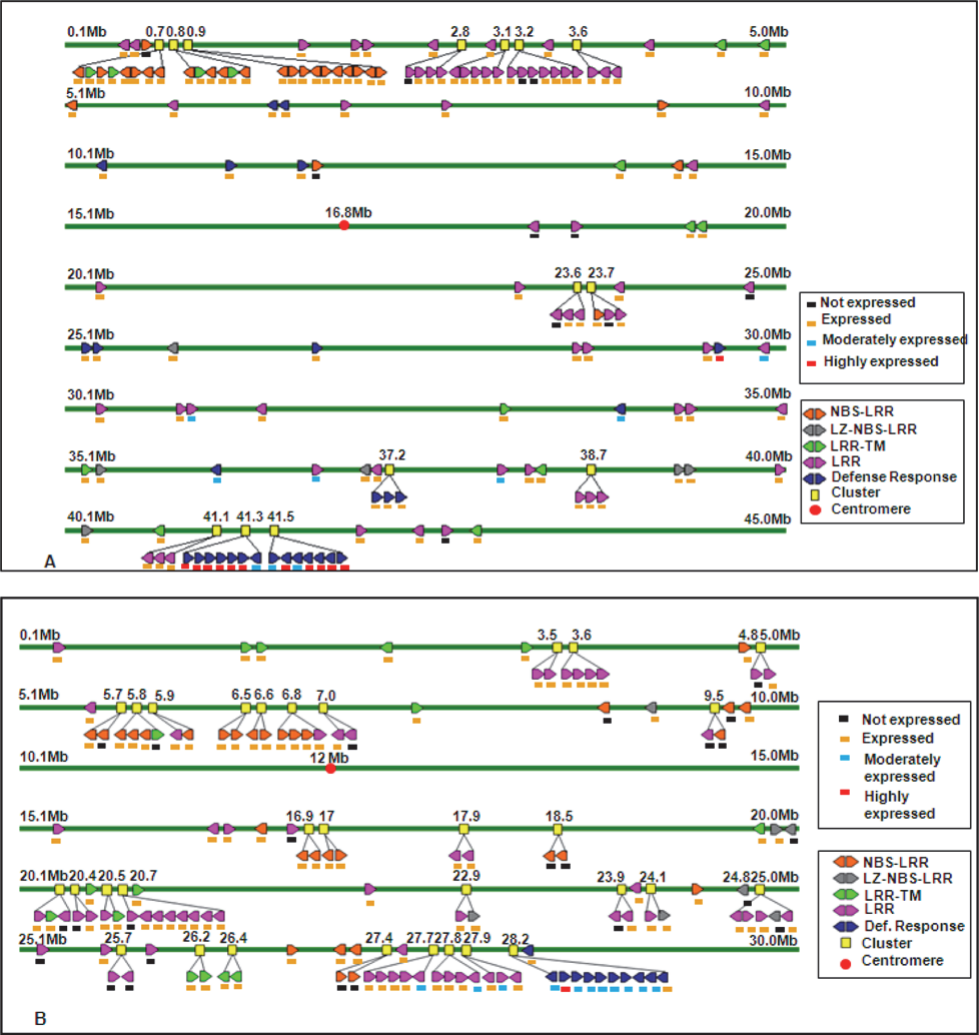
Mapping of R- and DR-genes. Example of physical position, orientation and expression of R- and DR-genes on *japonica* rice chromosomes. Representation of R- and DR-gene clusters mapped on chromosome 1(A) and chromosome 11(B). Arrow heads of genes indicate their forward and reverse orientation. Rectangles against each gene showed their expression level based on EST matches. Position of each cluster in terms of Mb is given on the top of each line representing chromosomal segments.

We analyzed clustering and orientation of all the genes present on different chromosomes of *japonica* rice and found 186 R- and DR-gene clusters. Our analysis showed that most of the R-genes were present in large clusters on all the chromosomes (S1–S10 Figs). An example of tandemly repeated genes identified on short arm of chromosome 1 is shown in Fig 3A. We found maximum (31) gene clusters in chromosomes 11 consisting of 92 genes (Fig 3B) followed by chromosome 1 which has 91 genes in 24 clusters. Number of clusters found in long arm and short arm of chromosome 1 were almost equal but the clusters found on short arm were large as compared to long arm. Similarly in chromosome 11, number of clusters found on long arm is just double the clusters found in short arm of the chromosome. The long arm of this chromosome consisted mostly of defense response genes and a big cluster of 11 tandemly repeated genes was identified. Similarly, gene clusters were identified in all the chromosomes of rice, indicating their origin by duplication from a few ancestral genes (Table 1). Many small and medium sized clusters were identified in rest of the chromosomes.

**Table 1:** Distribution of clusters of resistance gene and defense response genes over the twelverice chromosomes.

***O*. *sativa ssp*. *japonica***									
**Chr. No.**	**NBS-LRR**	**LZ-NBS-LRR**	**LRR-TM**	**LRR**	**DR**	**Mixed**	**Total clusters**	**Genes in clusters(%)**	**Total genes**
**1**	1	1	1	12	5	4	24	91(63.6)	143
**2**	2	1	0	4	0	10	17	47(49.5)	95
**3**	0	0	1	2	5	5	13	29(39.1)	74
**4**	2	0	0	6	1	0	9	23(44.2)	52
**5**	1	0	2	3	4	3	13	39(52.7)	74
**6**	4	0	0	3	3	9	19	52(65.8)	79
**7**	1	0	5	1	0	4	11	34(46.6)	73
**8**	0	1	0	4	1	7	13	36(48.0)	75
**9**	0	1	6	0	2	3	12	27(51.9)	52
**10**	0	1	1	3	0	3	8	23(48.9)	47
**11**	7	0	2	12	1	9	31	92(73.0)	126
**12**	5	0	1	4	2	4	16	37(58.7)	63
**Total**	**23**	**5**	**19**	**54**	**24**	**61**	**186**	**530(55.6)**	**953**
***O*. *sativa ssp*. *indica***									
**1**	1	1	0	9	4	13	28	89(69.5)	128
**2**	1	0	1	6	0	10	18	47(55.9)	84
**3**	0	0	0	5	2	7	14	33(43.3)	76
**4**	1	0	0	4	1	2	8	18(39.1)	46
**5**	0	0	1	2	1	8	12	38(55.8)	68
**6**	2	0	0	6	2	10	20	46(74.1)	62
**7**	1	0	3	2	1	7	14	37(57.8)	64
**8**	1	0	0	4	2	2	9	25(40.9)	61
**9**	0	0	4	2	2	5	13	27(56.2)	48
**10**	0	0	0	3	0	5	8	20(60.6)	33
**11**	1	0	1	7	3	12	24	85(83.3)	102
**12**	2	0	1	2	1	4	10	28(66.6)	42
**Total**	**10**	**1**	**11**	**52**	**19**	**85**	**178**	**493(60.5)**	**814**

The number of genes in each cluster ranged from 2 to 11. The highest percentages (44.3%) of genes were found in defense response category of gene clusters followed by NBS-LRR (43%) categories of gene clusters. Out of 396 genes in LRR category, 157 were present in clusters (Table 2). Most of these clusters were distributed over chromosomes 1 and 11. The number of gene clusters on chromosome 11 was the largest one including 92 tandemly repeated genes. In whole japonica rice genome, 55.6% of the R- and DR- genes are present in clusters.

**Table 2:** Distribution of different categories of resistance genes and defense response genes present in clusters in rice genome.

*O. sativa ssp. Japonica*															
S.No.	Cluster category	Rice chromosomes												Genes in cluster(%)	Total genes
		1	2	3	4	5	6	7	8	9	10	11	12		
**Resistance genes (No.)**															
**1**	**NBS-LRR**	10	4	0	5	2	12	2	0	0	0	14	12	61(43)	142
**2**	**LZ-NBS-LRR**	2	2	0	0	0	0	0	2	2	3	0	0	11(21)	53
**3**	**LRR-TM**	2	0	2	0	4	0	14	0	14	4	4	2	46(24)	195
**4**	**LRR**	38	11	4	16	10	9	3	12	0	8	38	8	157(40)	396
**5**	**Mixed**	19	30	12	0	12	25	15	19	7	8	26	8	181	
**Defense response genes (No.)**															
		20	0	11	2	11	6	0	3	4	0	10	7	74(44.3)	167
**Grand Total**		**91**	**47**	**29**	**23**	**39**	**52**	**34**	**36**	**27**	**23**	**92**	**37**	**530(55.6)**	**953**
***O*. *sativa ssp*. *Indica***															
**Resistance genes (No.)**															
**1**	**NBS-LRR**	2	2	0	4	0	4	2	2	0	0	3	7	26(22)	115
**2**	**LZ-NBS-LRR**	2	0	0	0	0	0	0	0	0	0	0	0	2(5)	43
**3**	**LRR-TM**	0	4	0	0	2	0	6	0	8	0	2	2	24(14)	166
**4**	**LRR**	25	15	13	8	4	15	5	8	4	6	24	5	132(38)	348
**5**	**Mixed**	47	26	16	4	28	23	22	10	10	14	48	11	259	
**Defense respone genes (No.)**															
		13	0	4	2	4	4	2	5	5	0	8	3	50(35)	142
**Grand Total**		**89**	**47**	**33**	**18**	**38**	**46**	**37**	**25**	**27**	**20**	**85**	**28**	**493(60.5)**	**814**

We analyzed clustering and orientation of all the genes present on different chromosomes of *indica* rice and found 178 R- and DR-gene clusters. Our analysis showed that most of the R-genes were present in large clusters on all the chromosomes. We found maximum (28) gene clusters in chromosomes 1 consisting of 89 genes followed by chromosome 11 which has 85 genes in 24 clusters (Table 1). Similarly, gene clusters were identified in all the chromosomes of rice, indicating their origin by duplication from a few ancestral genes (Table 1). Many small and medium sized clusters were identified in rest of the chromosomes.

The number of genes in each cluster ranged from 2 to 11. The highest percentages (38%) of genes were found in LRR category of gene clusters followed by defense response (35%) categories of gene clusters. Out of 115 genes in NBS-LRR category, 26 were present in clusters (Table 2). Most of these clusters were distributed over chromosomes 1 and 11. The number of gene clusters on chromosome 1 was the largest one including 89 tandemly repeated genes. In whole *indica* rice genome 60.5% of the R- and DR- genes are present in clusters.

We could not analyzed clustering and orientation of all the genes present on different chromosomes of *O*.*brahyantha* because it had unfinished genome at the time of analysis and as a result pseudomolecules of chromosome were not available.

We found that many of the R-genes analyzed in present investigation are present in clusters. Presence of more number of genes and gene clusters at few positions in the chromosomes indicates that there are chromosomal hot spots in which genes reside. The long period of contact between rice and its pathogens on these specific positions of chromosomes may help in R- genes expansion by duplication and rearrangement during genome shuffling [43]. Distribution of R- and DR-genes in cluster might provide a reservoir of genetic variation from which new variants of R-genes arises.

Botella et al. [44] reported earlier that clusters of disease-resistance and defense-response genes are found in the Arabidopsis genome [45]. Rice chromosome 11 has several large clusters of fast evolving disease resistance and defense response genes that might have originated by the process of tandem duplication and subsequent divergence under the selective pressure of rice pathogens [39]. The clustering of NBS-encoding genes in the rice genome might be because of these localized duplications as is also observed in the Arabidopsis genome [46, 18, 47] and inferred by mapping experiments in other species. The most promising feature of the clustering in the rice genome is the diversity of different genes within a cluster and the lack of large homogenous array of genes. Many of the clusters are composed of genes belonging to different classes. Rice also carries clusters of closely related genes, but they typically are small or have divergent members [19]. It has been reported that the genes in the *Rp1* and *Rp3* complexes of maize typically code for proteins with approximately 90–99% sequence identity. Largest cluster of well characterized disease resistance genes known in plants has been reported in lettuce [48]. Localized duplications of R-genes sometimes invert the orientation of the genes on that locus. This type of duplication also allows the genes to evolve more independently and diverge from other members of the cluster. It has been demonstrated by genetic mapping approaches that R-genes tend to be clustered in few chromosomes in the genome [49, 50]. In *A*. *thaliana*, these were reported to be clustered in two chromosome arms [41], similar pattern was obtained in rice [40]. The same R-genes have been found clustered and almost in the same order in tomato [51] and chickpea [50]. It confirms that gene order and proximity are important for the functional nature of these genes. Similar to the present investigation, most NBS-LRR genes are unevenly located in the plant genomes and are found in multigenes clusters. R-genes are quite abundant in higher plants, with 210 clusters found in FOREST database of Eucalyptus presenting significant homology to known R-genes [39]. Using 30 well-known R-genes as template, 196 clusters have been identified in SUCEST database [52]. All five classes of R-genes with their respective conserved domains have been found in sugarcane except the TIR domain which is not present in rest of the monocots previously studied [38].

### Identification of InDels within R- & DR-genes

Out of 186 clusters in *japonica* rice, 13 clusters were analyzed for the identification of insertions and deletions which might be accounting for phenotype of the genes. Gene prediction was done for 97 genes present in all the clusters of *japonica* and numbers of exons were determined. Coding sequences from each cluster were separately aligned using bioedit software. Diagrammatic representation of number of insertions and deletions at specific position of the genes in one of the clusters of chromosome 1 is shown in S11 Fig. Most of the InDels were 3 to 4 bases barring few exceptions. Out of 13 clusters (S2 Table), only 3 clusters have more number of deletions as compared to insertions. Largest (48 nt) deletion was found in the gene present in cluster 7 and a large insertion of 130 nt was found in the genes of cluster 5 (S3 Table). Detailed analysis of all the clusters revealed that number of insertions (82) was twice than that of number (41) of deletions (S4 Table).

In this study of identification of InDels within R- and DR-gene clusters of *O*. *sativa* spp *japonica* of rice we found more number of insertions as compared to number of deletions. These InDels might be responsible for changing the phenotype of genes in clusters. These indels may also contribute to the formation of pseudogenes. In potato, 39.4% of many NBS-LRR genes are predicted to be pseudogenes, because of the presence of indels [53]. Multiple LRRs resulted from unequal crossing over [54] within or between *RGC2* genes in lettuce. Similarly *indels* have been found in *RPP13* homologues in *Arabidopsis* and *Mla* genes in barley [55, 56].

### Analysis of evolutionary relationships among R- & DR-genes

Phylogenetic tree constructed for 97 genes of the genome of *Japonica* type rice line consist of 9 main clusters (S12 Fig). Most of the genes related to a specific class shared same cluster. Cluster 6 was the largest having 23.71% of the total number of R-gene followed by cluster 7 having 19.5% of the total numbers of R-genes and only 1% of the genes shared cluster 1 and cluster 2. The Ka/Ks values, which determine the non synonymous and synonymous amino acid substitution per site, were also calculated for each gene. For most of the genes, Ka/Ks value was more than one (S13 Fig). Out of 97 genes present in different clusters, 68 genes have more than one Ka/Ks. Therefore, the non synonymous substitution rate was more than synonymous substitution rate in R-genes and DR-genes of rice.

**Fig 4:**
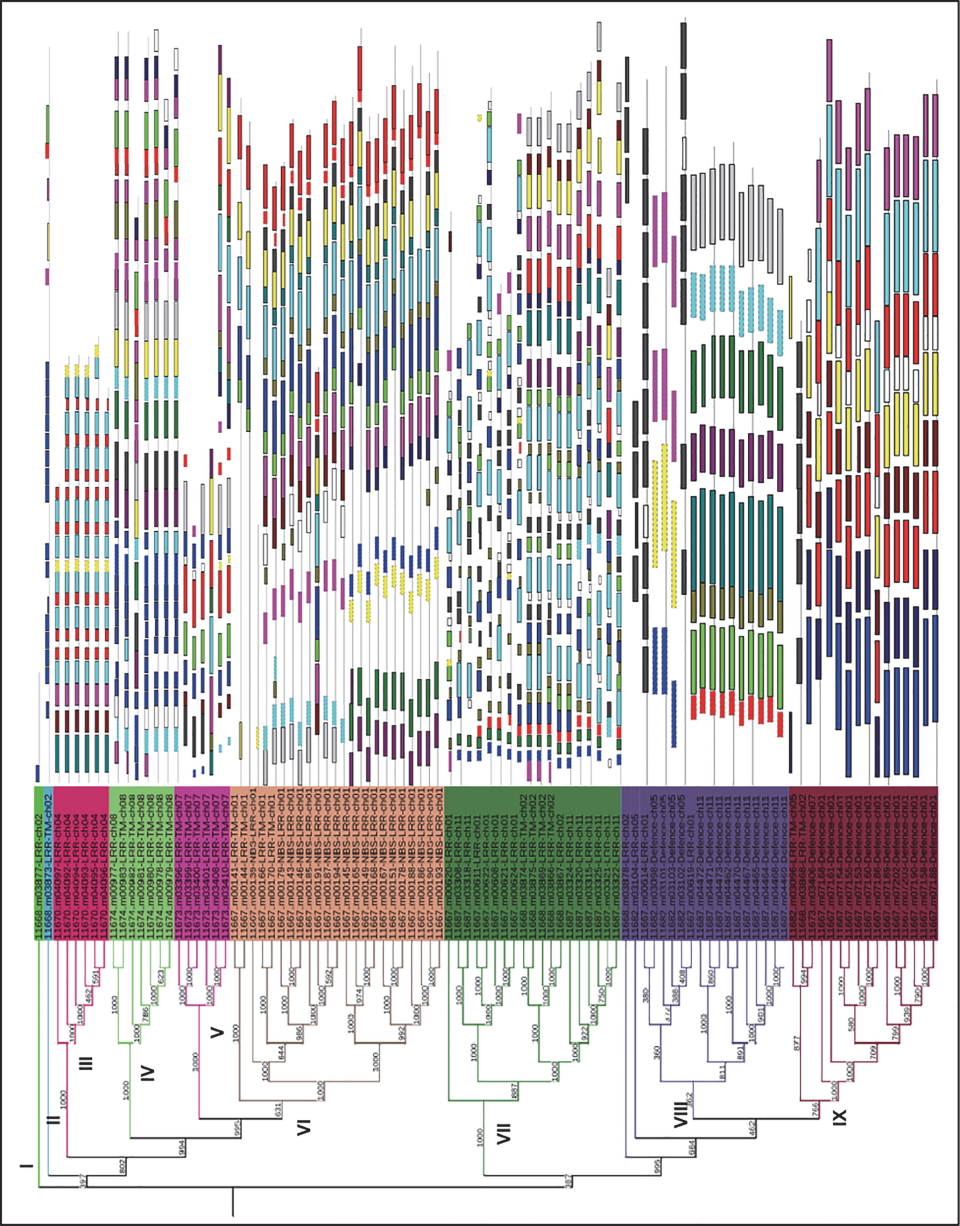
Phylogenetic and motif analysis of R- and DR-genes in clusters in *O*. *sativa ssp japonica*. The overlay of phylogenetic analysis and motif analysis of R-genes and DR-genes in clusters of 6 or more than 6 genes over 12 chromosomes of *O*. *sativa ssp japonica*. MEME 4.6.1 software was used following the parameters described in Method. Twenty conserved motifs were shaded in different colours. Several subgroups were distinguished by the motif distribution, which is consistent with the phylogenetic subgroups in all R- and DR-genes in clusters.

We carried out the motif analysis of R-genes and DR-genes of *japonica* present within the clusters (Fig 4). Motif search revealed 6 to 50 residues in all the genes (S5 Table). A correlation between the motif pattern and the phylogenetic tree was found, since each cluster shared the same motif pattern. Some other motifs were more specific to one class or sub class of R-genes. This study focuses on the fact that if genes are in clusters and phylogenetically they are in some clade then they may differ because of indels. Motif finding and phylogenetic analysis of the genes in each cluster in our investigation clearly depicted deep evolutionary origin of R-genes and DR-genes. The motif distribution indicated that the genes containing the same motifs might arise from gene expansion within the same class. The ancestor genes with various motif structure seem to appear early in the evolution, and such structure have been maintained through the evolution.

**Fig 5:**
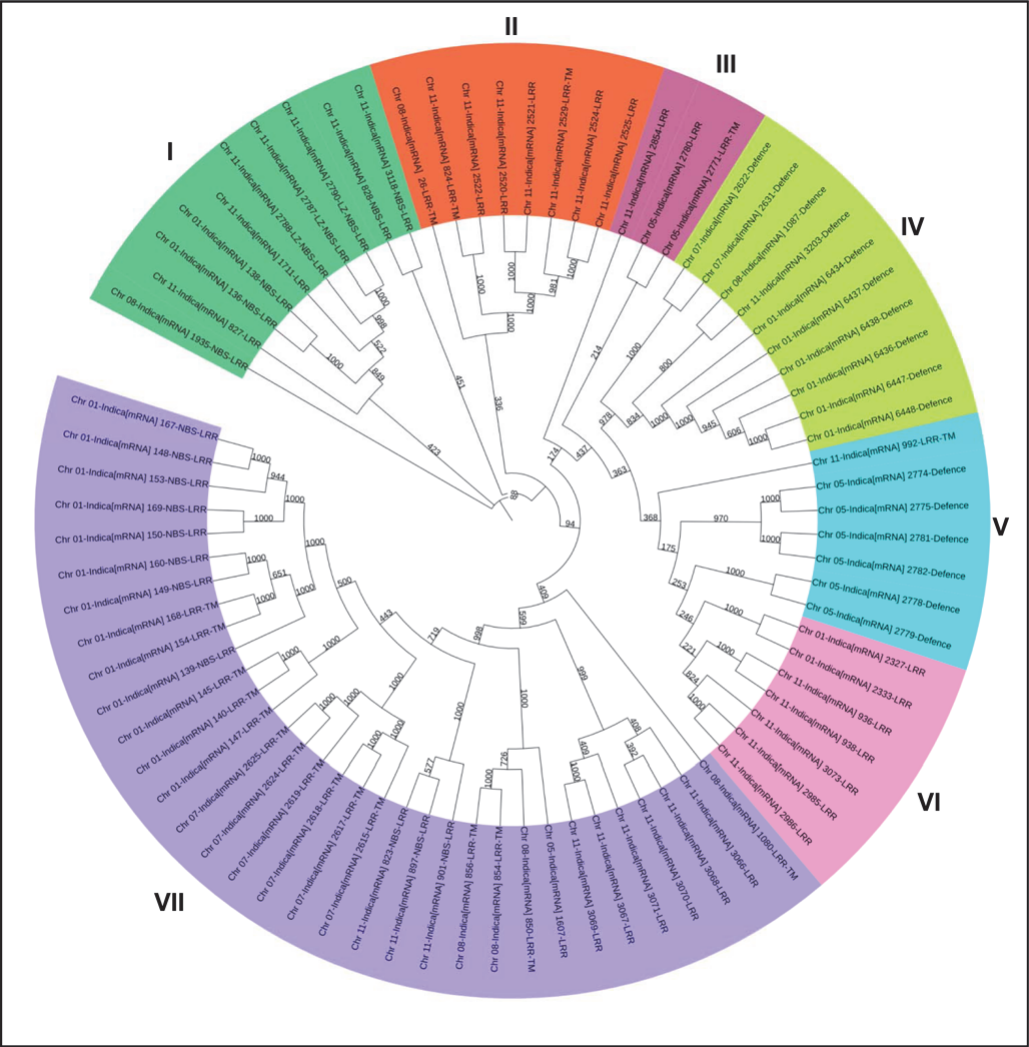
Phylogenetic analysis of R- and DR-genes in clusters in *O*. *sativa ssp indica*. Phylogenetic analysis of R-genes and DR-genes in clusters of 6 or more than 6 genes over 12 chromosomes of *O*. *sativa ssp indica*.

For *indica* type genome, phylogenetic analysis was performed for all the genes in each cluster. Phylogenetic tree constructed for 78 genes consist of 7 main clusters (Fig 5). Each group is further divided into sub groups, all of which were supported by high bootstrap values. Cluster 7 was the largest having 42% of the total number of R-gene followed by cluster 1 and cluster 4 having 12.8% of the total numbers of R-genes and only 3.8% of the genes shared cluster 3. It has also been reported that many of the closely linked gene clusters arose by divergence from one or a few progenitor genes and the duplications that invert orientation of the genes may promote divergence by inhibiting recombination [19].

Phylogenetic tree constructed for 167 DR genes consist of 6 main groups (S14 Fig). Each group was further divided into sub groups, all of which were supported by high bootstrap values. Each group was a mix of glucanases, chitinases and thaumatin-like genes. Group 5 devoid of thaumatin-like genes and had only chitinases and glucanases genes. Highest number of glucanases was found in group 4, though each group predominantly contained this type of genes. Group 4 was the largest having 32% of the total number of DR-genes and only 5.4% of the genes shared group 5. Phylogenetic tree performed for all DR genes showed that defense response genes were also found in closely related clusters. A large cluster of 11 chitinase genes identified on long arm of chromosome 11 has turned out to be a major QTL for resistance to Sheath blight fungus *Rhizoctonia solani* [57]. It showed clearly that *in silico* analysis can be correlated with typical genetic mapping of resistance genes and QTLs in rice.

The Ka/Ks value for glucanases, thaumatin-like genes and chitinases is shown in S15A Fig. For most of the genes Ka/Ks value was more than one (S15B Fig). In case of glucanases, out of 90 genes, 81 genes had more than one Ka/Ks value. Non synonymous substitution rate was more than the synonymous substitution rate in this class of DR-genes. For chitinases, out of 47 genes 35 had >1 Ka/Ks values whereas out of 29 thaumatin-like genes, 20 had >1 Ka/Ks values. In present study, ratio of synonymous and non synonymous substitution is more than one indicating positive selection and has more changes in amino acids. It has also been reported that most of the R-genes (>50%) in Arabidopsis were evolved under strong positive selection as characterized by high Ka/Ks ratios (>1), which is a major driver for generating interspecies variation in Arabidopsis R-genes [58].

**Fig 6:**
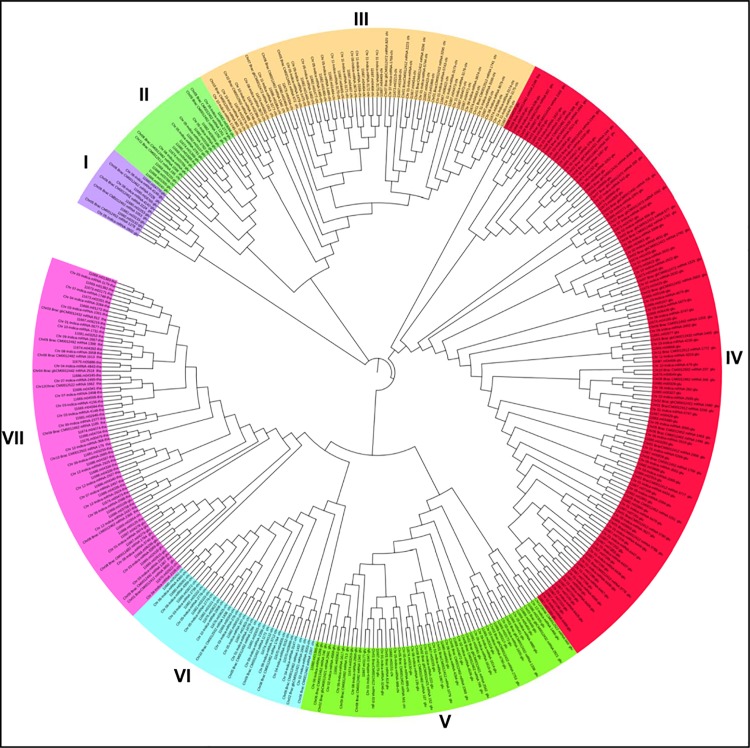
Phylogenetic tree for DR-genes. Phylogenetic tree constructed for different category of DR-genes for *O*. *sativa ssp japonica*, *O*. *sativa ssp indica* and *O*. *brachyantha*.

Phylogenetic tree constructed for 395 DR genes of *O*.*sativa*.*ssp*. *japonica*, *O*.*sativa ssp indica* and *O*. *brachyantha* consist of 7 main groups (Fig 6). Most of the genes related to a specific category shared same cluster. Cluster 1 consists of only glucanases. Highest number of glucanases was found in cluster 4 (134). Cluster 3 and cluster 6 consist of only chitinases. Cluster 4 was the largest having 34% of the total number of R-gene followed by cluster 7 having 18.2% of the total numbers of R-genes and only 3% of the genes shared cluster 1. Phylogenetic tree performed for all DR genes of three species showed that defense response genes of all three rice species were found in closely related clusters. Most of the clusters consisted of same category of DR-genes from all three species of rice. So it was concluded that phylogenetic tree obtained was divided on the basis of category of DR-genes and not on the basis of rice species which shows that DR-genes of all rice species are closely related.

### In silico expression pattern of R-genes and DR-genes

To validate computational gene prediction, expression analysis was studied by finding EST match to all the *in silico* identified genes in the database. All genes were classified into four categories like unexpressed, expressed, moderately expressed and highly expressed based on their significant hits against EST database.

**Fig 7:**
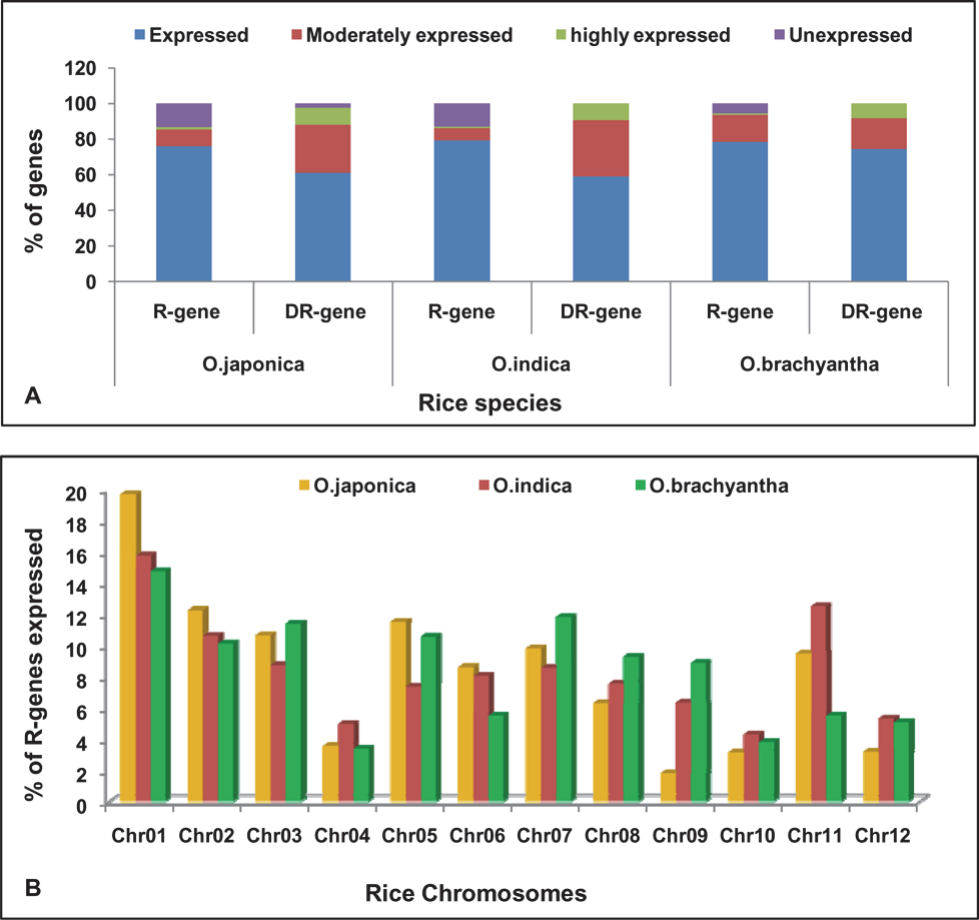
Expression pattern of R-like and DR-genes. (A)Percentage expression analysis of R-like and DR-genes in *O*. *sativa ssp japonica*, *O*. *sativa ssp indica* and *O*. *brachyantha* rice genomes. **(**B) Percentage of R-genes expressed and their distribution in each chromosome of *O*. *sativa ssp japonica*, *O*. *sativa ssp indica* and *O*. *brachyantha* rice genomes.

In *Japonica* line we found that 76%, 61% of the R-and DR-genes were expressed, 9.4%, 26.9% of the R- and DR-genes were moderately expressed and 1.1%, 9.5% of the R- and DR-genes were highly expressed, respectively (Fig 7A). More than 11% of the R-genes and 2.3% of the DR-genes were not expressed (pseudogenes) because they did not show any EST match. Maximum percentage (19.68%) of expressed R-genes was obtained on chromosome 1 followed by chromosome 2 (12.26%) whereas only 1.8% of the genes found expressed on chromosome 9 (Fig 7B).

**Fig 8:**
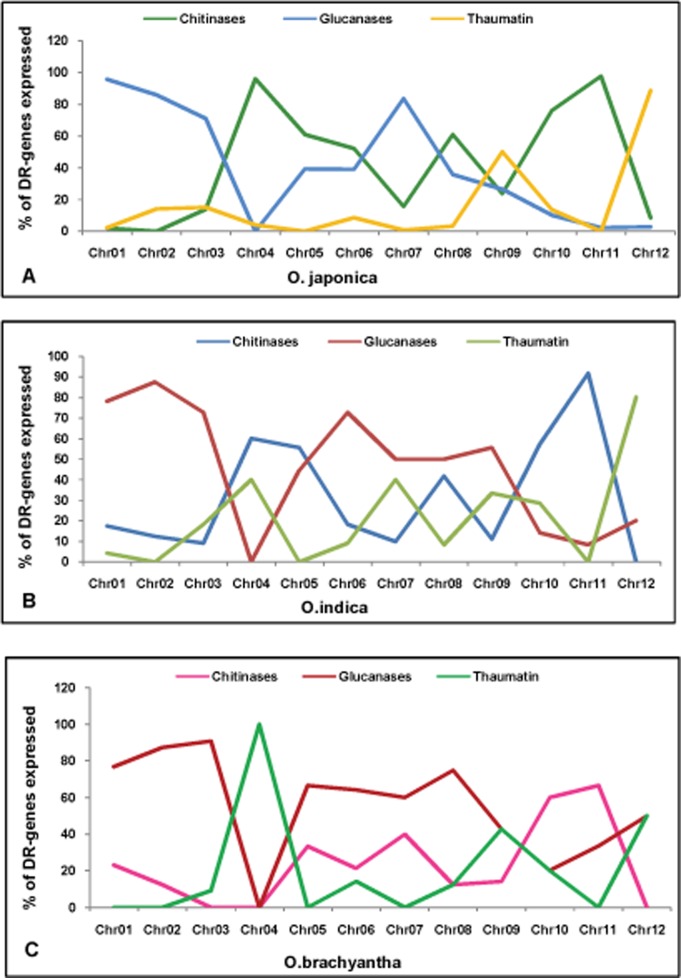
Expression analysis of classes of DR-genes. Expression analysis of different classes of defense response genes present on each rice chromosomes of (A) *O*. *sativa ssp japonica*, (B) *O*. *sativa ssp indica* and (C) *O*. *sativa ssp brachyantha*.

Among three categories of defense response genes, expression of glucanases was maximum (56.6%) on most of the chromosomes followed by chitinases (35.51%) and thaumatin (7.87%) like genes. Glucanases (95.77%), chitinases (97.62%) and thaumatin-like genes (50%) had shown maximum expression on chromosomes 1, 11 and 9, respectively (Fig 8A).

In *Indica*, we found that 79%, 59% of the R-and DR-genes were expressed, 6.8%, 36.8% of the R- and DR-genes were moderately expressed and 0.75%, 9.1% of the R- and DR-genes were highly expressed respectively (Fig 7A). More than 11% of the R-genes were not expressed (pseudogenes). All the DR-genes were expressed. Maximum percentage (15.75%) of expressed R-genes was obtained on chromosome 1 followed by chromosome 2 (10.6%) whereas only 4.2% of the genes found expressed on chromosome 10 (Fig 7B).

Among three categories of defense response genes, expression of glucanases was maximum (53.5%) on most of the chromosomes followed by chitinases (30.9%) and thaumatin (15.4%) like genes. Glucanases (87.5%), chitinases (91.6%) and thaumatin-like genes (80%) had shown maximum expression on chromosomes 2, 11 and 12, respectively (Fig 8B).

In *O*. *brachyantha*, we found that 78.4%, 74.4% of the R-and DR-genes were expressed, 15.1%, 17.4% of the R- and DR-genes were moderately expressed and 0.79%, 8.1% of the R- and DR-genes were highly expressed, respectively (Fig 7A). More than 5% of the R-genes were not expressed (pseudogenes). Maximum percentage (14.7%) of expressed R-genes was obtained on chromosome 1 followed by chromosome 7 (11.8%) whereas only 3.3% of the genes found expressed on chromosome 4 (Fig 7B).

Among three categories of defense response genes, expression of glucanases was maximum (66.2%) on most of the chromosomes followed by chitinases (22%) and thaumatin (11.6%) like genes. Glucanases (90%), chitinases (66.6%) and thaumatin-like genes (100%) had shown maximum expression on chromosomes 3, 11 and 4, respectively (Fig 8C). So expression analysis of R- and DR-genes of three species concludes that percentage expression of R-genes of brachyantha was more than percentage expression of *indica* and *japonica* and for DR-genes, only one DR-gene of *japonica* was not expressed while for *indica* and *brachyantha* all DR-genes were expressed.

The analysis of finding expression of all identified defense and disease resistance genes on the basis of already known and expressed genes in KOME database concluded that about 11.43% of the genes were not expressed and might be pseudogenes. Meyers et al. (2003) found that nearly 10% of the NBS-LRR genes in the Columbia ecotype of Arabidopsis were apparent pseudogenes. Another report claimed that approximately 20% of the NBS-LRR genes in Nipponbare genome were predicted to be pseudogenes [19]. The pseudogenes are those non functional copies of the genes, which were created by genomic duplication. Among defense response genes, glucanases have shown maximum expression. Only one DR gene of *japonica* was pseudogene and rest were expressed indicating that defense response genes act in a coordinated manner against the incoming plant pathogens.

### Identification of paralogous genes

**Fig 9:**
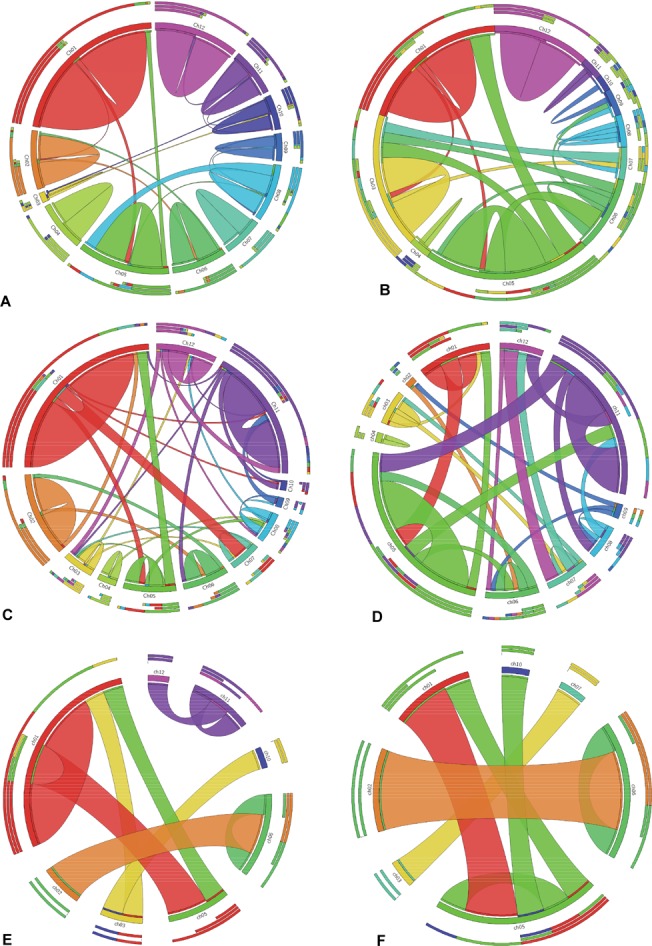
R- and DR-genes paralogs. Number of R- and DR-genes paralogs present in three rice genomes using CIRCOS software. (A) Paralogs of R-genes in *O*. *sativa ssp japonica*, (B) Paralogs of DR-genes in *O*. *sativa ssp japonica*, (C) Paralogs of R-genes in *O*. *sativa ssp indica*, (D) Paralogs of DR-genes in *O*. *sativa ssp indica*, (E) Paralogs of R-genes in *O*. *brachyantha*, (F) Paralogs of DR-genes in *O*. *brachyantha*. The circle represents rice chromosomes having paralogous genes and their number of paralogous gene matches on different rice chromosomes.

**Fig 10:**
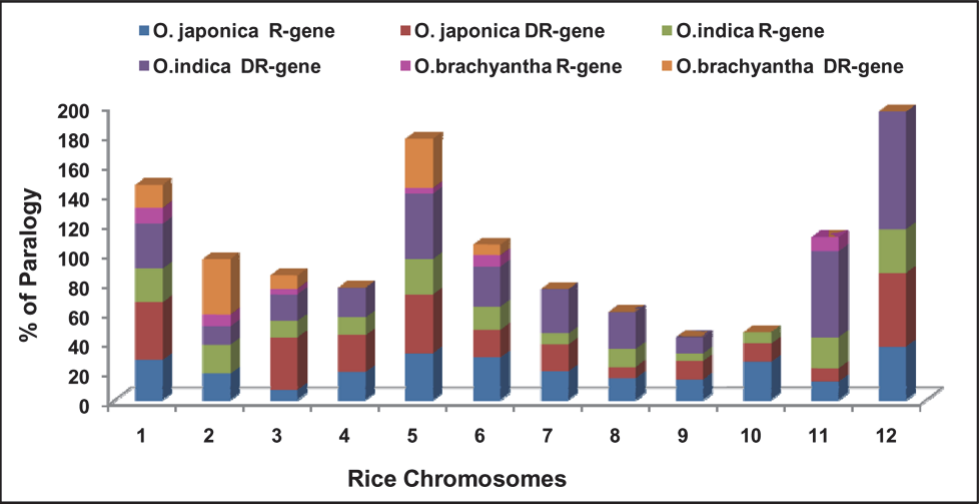
Percentage of paralogy. Percentage of paralogy for R- and DR-genes in twelve rice chromosomes of *O*. *sativa ssp japonica*, *O*. *sativa ssp indica* and *O*. *brachyantha*.

Paralogs of R- and DR-genes were identified using the genes already annotated in present study. In *japonica* rice genome, 20.7%, 26.3% paralogues were found for R-genes and DR-genes, respectively. Out of 786 R-genes, only 163 R-genes were found to have 278 paralogs in the genome. However, 44 DR-genes showed presence of 61 paralogs (S6 Table). The paralogs of R- and DR-genes were studied separately for each chromosome of rice. For R-genes more than 90% paralogs were found on same chromosome and only 8% on different chromosomes (Fig 9A) but for DR-genes 30% of paralogs were found on different chromosomes and rest (70%) in the same chromosome (Fig 9B). The maximum numbers of paralogous (65) were found for R-genes on chromosome 1. Whereas for DR-genes maximum paralogous genes (15) were found on chromosome 3 (S6 Table). However, maximum percentage of paralogy for R- and DR-genes was found on chromosome 12 (Fig 10). Total 339 R- and DR-genes are found in *japonica* rice genome which are paralogs of each other. Average number of paralogs per gene ranged from 1 to 8. It was found that during the course of evolution only 9.5% of R- and DR-genes have changed their function, rest of the genes maintained their identity.

**Fig 11:**
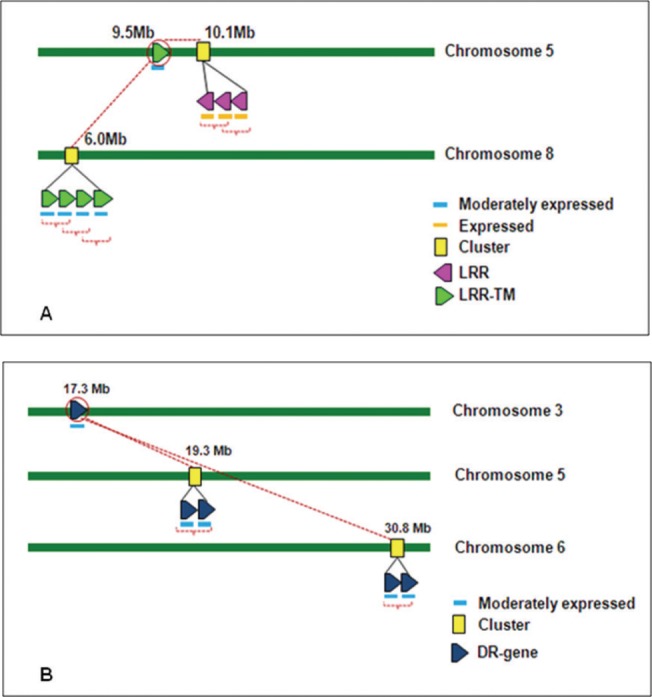
Interrelationship between R-genes and their paralogs present in cluster of *O*. *sativa ssp japonica*. An example of interrelationship between genes and their paralogs present in cluster of *O*. *sativa ssp japonica*. (A) The paralogs of an R-gene within and between chromosomes. Gene with outer red circle on chromosome 5 at 9.5 Mb is the gene for which paralogs were identified. Red dotted lines shows the paralogs of target gene on chromosome 5 and chromosome 8 at 10.1 Mb cluster and 6.0 Mb cluster, respectively. (B) The paralogs of a DR-gene between different chromosomes. Gene with outer red circle on chromosome 3 at 17.3 Mb is the gene for which paralogs were identified. Red dotted lines shows the paralogs of target gene clusters on chromosome 5 and chromosome 6 at 19.3 Mb and 30.8 Mb, respectively. All genes in clusters are also paralogs to each other.

To understand whether tandem gene duplication which affect gene clusters also effect paralogs of R- and DR-genes and their evolution, therefore, separate study of the clustered paralogs was also conducted. This study showed that more than 80% paralogous genes belong to the gene clusters (S6 Table). An example of inter-relationship between genes and their paralogs in cluster across chromosomes is given in Fig 11. In this example, one of the genes present on chromosome 5 at 9.5Mb has its three paralogs at 10.1 Mb on the same chromosome and also on chromosome 8 at 6.0 Mb position where the gene was duplicated in four copies (Fig 11A). All the copies of these genes are expressed. In another interesting example one of the defense response gene found at 17.3 Mb position has its paralogs at chromosome 5 and chromosome 6 at different positions (Fig 11B). Similar types of diverse distribution of paralogs on different chromosomes were found in the rice genome (S6 Table). The expression analysis of R-gene paralogs showed that 94% of these were expressed genes. The expression analysis of DR-gene paralogs showed more number of highly expressed genes as compared to R-gene paralogs (S6 Table).

In *Indica* genome, 16.9%, 29.5% paralogues were found for R-genes and DR-genes respectively. Out of 672 R-genes, only 114 R-genes were found to have 170 paralogs in the genome. However, 42 DR-genes showed presence of 69 paralogs (S6 Table). The paralogs of R- and DR-genes were studied separately for each chromosome of rice. For R-genes more than 70% paralogs were found on same chromosome and only 28% on different chromosomes (Fig 9C) but for DR-genes 49.2% of paralogs were found on different chromosomes and rest (50.8%) in the same chromosome (Fig 9D). The maximum number of paralogous (48) were found for R-genes on chromosome 1. Whereas for DR-genes, maximum paralogous genes (18) were found on chromosome 5 (S6 Table). However, maximum percentage of paralogy for R- and DR-genes was found on chromosome 12 (Fig 10). Total 239 R- and DR-genes are found in rice genome which is paralogs of each other. Average number of paralogs per gene ranged from 1 to 8. Study of clustered paralogs showed that more than 78% paralogous genes belong to gene clusters (S6 Table). The expression analysis of R-gene paralogs showed that 87.7% of these were expressed genes and fourteen genes were not expressed. The expression analysis of DR-gene paralogs showed more number of highly expressed genes as compared to R-gene paralogs (S6 Table).

In *O*. *brachyantha* rice genome, 4.38%, 11.6% paralogues were found for R-genes and DR-genes respectively. Out of 251 R-genes, only 11 R-genes were found to have 13 paralogs in the genome. However, 10 DR-genes showed presence of 10 paralogs (S6 Table). The paralogs of R- and DR-genes were studied separately for each chromosome of rice. For R-genes only 38.5% paralogs were found on same chromosome and 61.5% on different chromosomes (Fig 9E) but for DR-genes 70% of paralogs were found on different chromosomes and rest (30%) in the same chromosome (Fig 9F). The maximum number of paralogous (5) were found for R-genes on chromosome 1. Whereas for DR-genes maximum paralogous genes (3) were found on chromosome 5 and 2 (S6 Table).). However, maximum percentage of paralogy for R- and DR-genes was found on chromosome 1 and chromosome 2, respectively (Fig 10). Total 23 R- and DR-genes are found in rice genome which are paralogs of each other. Average number of paralogs per gene ranged from 1 to 2. The expression analysis of R-gene paralogs showed that more than 91% of these were expressed genes and one genes were not expressed. The expression analysis of DR-gene paralogs showed more number of highly expressed genes as compared to R-gene paralogs (S6 Table). All the paralogs of DR-gene were found expressed. Therefore present study on paralogy analysis provides useful insight into the way genomes evolves and about gene duplication events.

In eukaryotes gene duplication is a common phenomenon. As expected many genes which gets duplicated due to whole genome duplication are more similar to one another. The gene duplication results in the formation of more copies of the genes, which are known as paralogs. Rice paralogs have been studied for their characterization, expression and evolution [59, 60, 61]. In present study, analysis of paralogs was performed to know the copies of R- and DR-genes distributed on all rice chromosomes. Most of the paralogs in japonica and indica were found in the parent chromosome showing less shift of genes across the chromosomes whereas in O. brachyantha the result is just opposite showing more gene shift across chromosomes. The gene duplication is known as a primary source of genetic material available for evolution of the genes with new functions [62].

### Identification of orthologous genes

**Fig 12:**
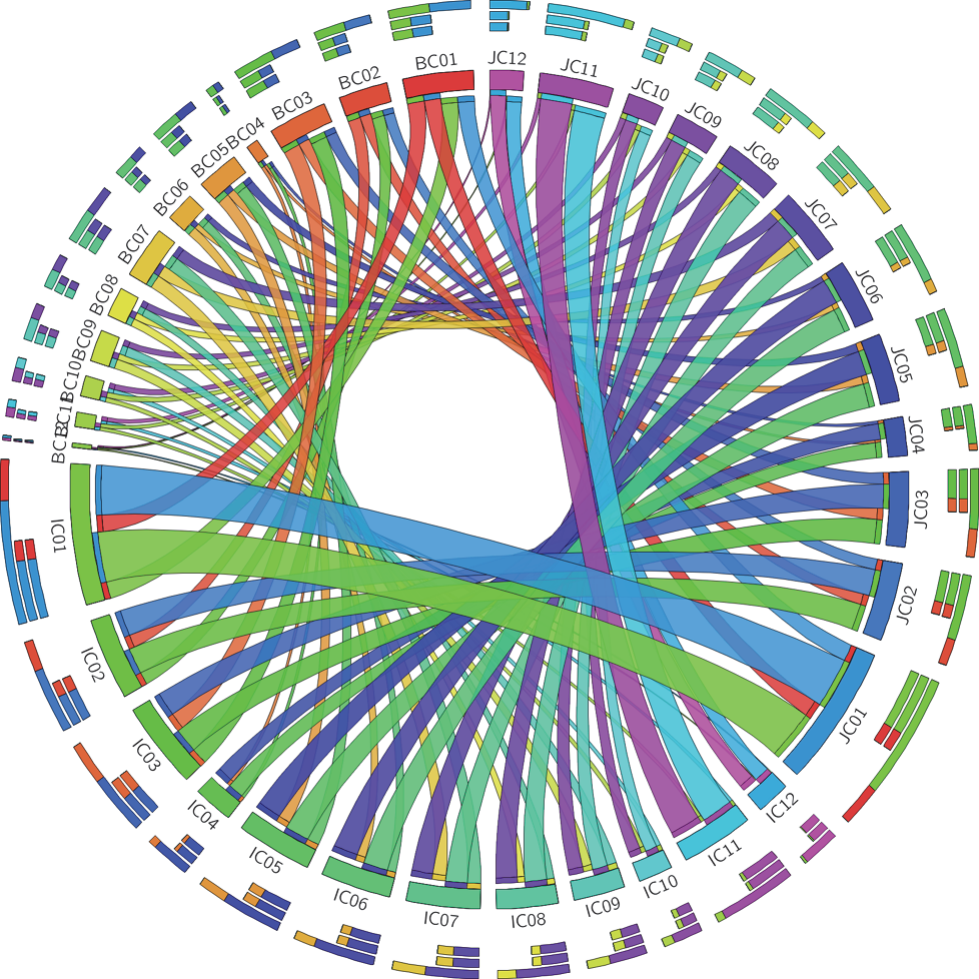
Orthologs for three rice genomes. Number of orthologous pairs for R- and DR-genes present between three rice species (*O*. *sativa ssp japonica*, *O*. *sativa ssp indica* and *O*. *brachyantha*) using CIRCOS software. Each ribbon arising from a species corresponds to the number of orthologous pairs with the destined species. The circle represents rice chromosomes having orthologous gene pairs and their number of orthologous gene matches on different rice chromosomes.

Numbers of orthologous pairs were found for R- and DR-genes between three rice species as described in the methods. The orthology analysis revealed that for R- and DR-genes, *O*. *brachyantha* has the least orthology with *O*. *indica* and *O*. *japonica* (Fig 12). Each ribbon arising from specific chromosome for a species (shown as clades) corresponds to the number of orthologous pairs with the chromosome of destined species. For instance, R- and DR-genes of *O*. *brachyantha* chromosome 12 was clearly found to have the minimum orthology with other rice species (*O*. *japonica* and *O*. *indica*). Maximum orthology is shared between *O*. *indica* and *O*. *japonica*. Between *japonica* and *indica* maximum number of orthologous pairs (93) was found in chromosome 1 and least number (22) in chromosome 10. Between *brachyantha* and *indica* maximum number of orthologous pairs (31) were found in chromosome 1 and least number (2) in chromosome 12 and between *japonica* and *brachyantha* maximum number of orthologous pairs (31) were found in chromosome 1 and least number (2) in chromosome 12. So *brachyantha* shows minimum orthology with both *indica* and *japonica*.

## Conclusions

Using bioinformatics tools, it was possible to identify and classify R-genes in the rice genome and also to make some inferences regarding their evolution and expression pattern. The exact map positions of all the rice disease resistance and defense response genes identified in this study could therefore be very useful in predicting whether related genes correspond to resistance genes in other cereals by performing synteny analysis. In silico analysis of R-genes in the rice genome would be important for functional validation of R-genes and allele mining in different rice lines and land races. Structural organization of R-genes, gene paralogs and important allelic variants found in this study can be used after validation for developing gene specific DNA markers which can be used in resistance breeding.

## Materials and Methods

### Genomic resources used

Complete genome sequence of *Oryza sativa ssp*. *japonica* was downloaded from TIGR database (http://rice.plantbiology.msu.edu/) and that of *Oryza sativa ssp indica* and *Oryza brachyantha* from NCBI database (www.ncbi.nlm.nih.gov) and BGI database(ftp://ftp.genomics.org.cn/ pub/ricedb/SynVs9311/9311/Sequence/SupScaffold/).

### Identification and chromosomal distribution of R-genes and DR-genes

The rice pseudomolecule sequences (TIGR Release 6.1, June 2009) were downloaded (http://rice.plantbiology.msu.edu/) [63] and a local database was created at the National Research Centre on Plant Biotechnology. The TIGR cDNA sequences were also retrieved chromosome wise and split into batches. These batches were subjected to local BLASTN against the local database of R_gene_ESTs. The BLASTN search parameters were optimized as-G 5,-E 1,-q -1,-r 1,-v 1,-b 1. The optimized BLAST search parameters were used in comparative analysis.

These BLAST results were tabulated into an excel file using an in-house developed Perl script “blast”. The similar process was repeated with local database of rice pseudomolecules for significant filtered results (Bit score >200, e-value ≤ 0) to know their positions on the rice pseudomolecules. The output file was searched with different keywords/ phrases using auto filters to represent R-like and defense response genes, and categorized into five main classes as follows: (i) NBS-LRR (matching with NBS-LRR, but not with LZ-NBS-LRR and LRR, CC-NBS-LRR, *Pib*, *Pita*, *Rp 1-d8*, *Lr10*, *Mla 1* and rust resistance), (ii) LZ-NBS-LRR (matching with LZ-NBS-LRR, but not with NBS-LRR, CC-NBS-LRR, LRR and RPM1), (iii) LRR-TM (matching with *Xa21*, serine/threonine kinases and Cf2/Cf5 resistance), (iv) LRR (matching with disease resistance, viral resistance, Yr10, LRR, but not with NBS-LRR, CC-NBS-LRR, LZ-NBS-LRR), (v) defense response genes (matching with glucanases, chitinases and thaumatin like genes) [40].

A list of predicted R-genes and DR-genes along with their physical position on pseudomolecules were compiled in an excel file. Then graphical presentation was made to locate the genes with orientation on each rice chromosome.

Whole genome sequence of each chromosome of *Oryza indica* was downloaded from ncbi.nlm.nih.gov and then gene prediction was done for each chromosome using Molquest software (www.molquest.com). The BLASTn was done for each gene of 12 chromosome with database (already identified 953 R- and DR-genes of *O*. *japonica*) to find out R- and DR-genes in *indica* genome. Blast result was filtered on the basis of bit score ≥200, e-value ≤ 0 and % identity ≥ 95% for R- and DR-genes and tabulated chromosome wise. Then blastX of R- and DR-genes obtained was done chromosome wise with downloaded superscaffolds of *O*. *indica* to know the position of genes on chromosomes.

Whole genome sequence of each chromosome of *Oryza brachyantha* was downloaded from ncbi.nlm.nih.gov and then gene prediction was done for each chromosome using Molquest software (www.molquest.com). The BLASTn was done for each gene of 12 chromosome with database (already identified 953 R- and DR-genes of *O*. *japonica*) to find out R- and DR-genes in *indica* genome. BLAST results were filtered on the basis of bit score ≥200, e-value ≤ 0 and % identity ≥ 95% for R- and DR-genes and tabulated chromosome wise. It was not possible to find out the position of each gene because *Oryza brachyantha* genome was unfinished at the time of analysis and pseudomolecules are not available.

### Multiple sequence and phylogenetic analyses of R- and DR-genes within clusters

Gene prediction was done for all 97 genes (*O*. *japonica*) in clusters using softberry software (www.softberry.com) and number of exons and their positions were determined. Coding sequences from each cluster were separately aligned using bioedit software version 7.0.8.0 [64] and consensus was generated. Number of insertion and deletions were found for each cluster. Multiple sequence alignment was performed for all genes in clusters using Clustalx software [65] and phylogenetic tree was generated. The Neighbour-joining method was used with the following parameters: pairwise deletion of gaps/missing data; bootstrap 1000 replicates and random seed of phylogeny test. The tree was viewed and edited using an online tool named iTOL (Interactive Tree Of Life) [66]. The sequence analysis for all R & DR genes in clusters was performed by using DnaSP 5.0 software (http://www.ub.es/DnaSP/DnaSP500html) [67] based on the Nei and Gojobori (1986) method [68] to calculate Ka/Ks values, which determine the non synonymous and synonymous amino acid substitution per site. We carried out the motif finding analysis to correlate motif with the phylogenetic analysis of R-genes and DR-genes in clusters using MEME 4.6.1 software [69] with the following parameters; distribution of motif occurrences: any number of repetitions; number of different motifs: 20; minimum motif width: 6; and maximum motif width: 50. For this analysis protein sequences of all genes in clusters were extracted. The height of the motif "block" is proportional to-log (p-value), truncated at the height for a motif with a p-value of 1e-10. The length of line in figure shows the length of a sequence relative to all other sequences. The position of a block shows where a motif has matched the sequence. The width of a block shows the width of the motif relative to the length of the sequence. The colour and border of a block identifies the matching motif as given in the legend. The height of a block gives an indication of the significance of the match as taller blocks are more significant. Some motifs were more conserved and present in most of the classes of genes. About twenty different motifs between 6 to 50 residues were detected by MEME 4.6.1 software [69]. The tree thus formed is found to be correlated and well supported. Similarly phylogenetic tree was constructed for R- and DR-genes in clusters for *O*. *sativa* ssp. *indica*. Such type of tree could not be formed for *O*. *brachyantha* because clusters of genes could not be obtained for them as exact positions could not be traced for R- and DR-genes.

### Phylogenetic analysis of DR-genes

Phylogenetic analysis was carried out for all types of defense response genes for *O*. *japonica*. This analysis was performed using MEGA4.1 software (http://www.megasoftware.net) [70]. The sequences were aligned by clustal W software (http://align.genome.jp) [71] and neighbor joining tree with p-distance, model was constructed using the tree drawing application in the MEGA4.1 [70]. For multiple sequence alignment, parameters like, Gap open penalty-15, Gap extension penalty-6.66 and weight matrix- IUB (for DNA) parameters were used. The confidence levels of nodes were tested by the bootstrapping of 1000 replications, and bootstrap values are indicated on the branches of tree. Phylogenetic tree was constructed with this software to study the evolution of defense response genes.

Substitution in nucleotides has great biological significance. Therefore, the value of Ks, synonymous (no change in amino) and Ka, non-synonymous (change in amino acid sequence) substitution was calculated. When positive selection dominates, the Ka/Ks ratio is greater than 1, means that diversity at the amino acid level is favored to the fitness advantage provided by the mutation. Conversely, when negative selection dominates, the Ka/Ks ratio is less than 1, means that most amino acid changes are deleterious. When the positive and negative selection forces balance each other, the Ka/Ks ratio is close to 1.These values were used for calculating Ka/Ks ratios among defense response genes by using DnaSP v5.0 software (http://www.ub.es/DnaSP/DnaSP500html) [67] based on the Nei and Gojobori (1986) method [68].

Phylogenetic analysis was carried out for all types of defense response genes for *O*.*sativa ssp*. *japonica*, *O*.*sativa ssp*. *indica* and *O*. *brachyantha* collectively. Multiple sequence alignment (MSA) was performed for all DR-genes to construct a phylogenetic tree by Clustalx software [65] using default parameters. The Neighbour-joining distance tree was constructed using default settings. The tree was viewed and edited using an online tool named iTOL (Interactive Tree Of Life) [66].

### Estimation of ESTs abundance for expression analysis

Rice EST sequences were retrieved from KOME database [72] and a local database was created at National Research Centre on Plant Biotechnology. The putative R- and DR-genes of *O*. *japonica* were subjected to BLAST against the local database of rice ESTs. The results were tabulated in an excel file and the number of significant hits (bit score ≥100 and E-value ≤ e^-20^) were calculated for each gene. On the basis of significant hits obtained, genes were classified into unexpressesed genes (no EST hits), expressed genes (1–100 hits), moderately expressed genes (101–400 hits) and highly expressed genes (>400 hits) [35]. The data were expressed in terms of percentage of total number of genes. The categorized genes of *O*.*sativa ssp*. *japonica* (in terms of expression) were used for the expression analysis in *O*.*sativa ssp*. *indica* and *O*. *brachyantha* genomes.

### Analysis of paralogous genes (gene duplication)

In this study, the paralogs were defined as two or more different genes in the same species which are so similar in their nucleotide sequences that they are assumed to have originated following the duplication of a single ancestral gene having significant hits of bit scores of >100, e values of <e−20, percent identity >80% and genome coverage >50%. All 786 R- and 167 DR-genes of *O*. *japonica* were separately BLAST searched against each other using default parameters. The BLAST search output was processed using BLAST Parser software (http://geneproject.altervista.org/). All the hits meeting this criteria for each of the twelve rice chromosomes was counted and tabulated using Microsoft Excel. Chromosomal positions of both R- and DR-genes were retained in the gene headers for the analysis. Comparison was made for each gene and a circular synteny map was plotted according to Krzywinski et al. (2009) [73]. Expression analysis of all paralogous R- and DR-genes was performed on the basis of already defined criteria in this section. The R- and DR-gene clusters were carefully examined to know the position of paralogous genes and to conclude the number of paralogous genes belonging to clusters. Similarly this analysis was done for *O*.*sativa ssp*. *indica* and *O*. *brachyantha* genomes respectively.

### Analysis of orthologous genes (synteny analysis)

Syntenic relationship was inferred between R- and DR-genes of three genomes by finding orthologs between them. For determining orthologs, we performed all against-all BLAST search of the genes on one genome against the other. Only significant hits meeting the criteria of BLAST bit score ≥100, E-value ≤ e-^20^ and 80% identity between gene sequences over at least 50% of the gene length were choosen for the analysis. If two significant BLAST hits match the above mentioned parameter and have bidirectional hits with each other, then they were considered as orthologs to each other and were counted as single orthologous pair [35]. The BLAST search output was processed using BLAST Parser software (http://geneproject.altervista.org/). The number of orthologous pairs were detected, tabulated in excel worksheets and then represented in the form of figure by Circos software [73].

## Supporting Information

S1 Fig Mapping of R- and DR-genes on *japonica* rice chromosome 2. Example of physical position, orientation and expression of R-genes and DR-genes on *japonica* rice chromosome 2. Arrow heads of genes indicate their orientation. Rectangles against each gene showed their expression level based on EST matches. Position of each cluster in terms of Mb is given on the top of each line representing chromosomal segments. Class miscellaneous in figure stand for LRR (Leucine Rich Repeat).
(TIF)

S2 Fig Mapping of R- and DR-genes on *japonica* rice chromosome 3. Example of physical position, orientation and expression of R-genes and DR-genes on *japonica* rice chromosome 3. Arrow heads of genes indicate their orientation. Rectangles against each gene showed their expression level based on EST matches. Position of each cluster in terms of Mb is given on the top of each line representing chromosomal segments. Class miscellaneous in figure stand for LRR (Leucine Rich Repeat).
(TIF)

S3 Fig Mapping of R- and DR-genes on *japonica* rice chromosome 4. Example of physical position, orientation and expression of R-genes and DR-genes on *japonica* rice chromosome 4. Arrow heads of genes indicate their orientation. Rectangles against each gene showed their expression level based on EST matches. Position of each cluster in terms of Mb is given on the top of each line representing chromosomal segments. Class miscellaneous in figure stand for LRR (Leucine Rich Repeat).
(TIF)

S4 Fig Mapping of R- and DR-genes on *japonica* rice chromosome 5. Example of physical position, orientation and expression of R-genes and DR-genes on *japonica* rice chromosome 5. Arrow heads of genes indicate their orientation. Rectangles against each gene showed their expression level based on EST matches. Position of each cluster in terms of Mb is given on the top of each line representing chromosomal segments. Class miscellaneous in figure stand for LRR (Leucine Rich Repeat).
(TIF)

S5 Fig Mapping of R- and DR-genes on *japonica* rice chromosome 6. Example of physical position, orientation and expression of R-genes and DR-genes on *japonica* rice chromosome 6. Arrow heads of genes indicate their orientation. Rectangles against each gene showed their expression level based on EST matches. Position of each cluster in terms of Mb is given on the top of each line representing chromosomal segments. Class miscellaneous in figure stand for LRR (Leucine Rich Repeat).
(TIF)

S6 Fig Mapping of R- and DR-genes on *japonica* rice chromosome 7. Example of physical position, orientation and expression of R-genes and DR-genes on *japonica* rice chromosome 7. Arrow heads of genes indicate their orientation. Rectangles against each gene showed their expression level based on EST matches. Position of each cluster in terms of Mb is given on the top of each line representing chromosomal segments. Class miscellaneous in figure stand for LRR (Leucine Rich Repeat).
(TIF)

S7 Fig Mapping of R- and DR-genes on *japonica* rice chromosome 8. Example of physical position, orientation and expression of R-genes and DR-genes on *japonica* rice chromosome 8. Arrow heads of genes indicate their orientation. Rectangles against each gene showed their expression level based on EST matches. Position of each cluster in terms of Mb is given on the top of each line representing chromosomal segments. Class miscellaneous in figure stand for LRR (Leucine Rich Repeat).
(TIF)

S8 Fig Mapping of R- and DR-genes on *japonica* rice chromosome 9. Example of physical position, orientation and expression of R-genes and DR-genes on *japonica* rice chromosome 9. Arrow heads of genes indicate their orientation. Rectangles against each gene showed their expression level based on EST matches. Position of each cluster in terms of Mb is given on the top of each line representing chromosomal segments. Class miscellaneous in figure stand for LRR (Leucine Rich Repeat).
(TIF)

S9 Fig Mapping of R- and DR-genes on *japonica* rice chromosome 10. Example of physical position, orientation and expression of R-genes and DR-genes on *japonica* rice chromosome 10. Arrow heads of genes indicate their orientation. Rectangles against each gene showed their expression level based on EST matches. Position of each cluster in terms of Mb is given on the top of each line representing chromosomal segments. Class miscellaneous in figure stand for LRR (Leucine Rich Repeat).
(TIF)

S10 Fig Mapping of R- and DR-genes on *japonica* rice chromosome 12. Example of physical position, orientation and expression of R-genes and DR-genes on *japonica* rice chromosome 12. Arrow heads of genes indicate their orientation. Rectangles against each gene showed their expression level based on EST matches. Position of each cluster in terms of Mb is given on the top of each line representing chromosomal segments. Class miscellaneous in figure stand for LRR (Leucine Rich Repeat).
(TIF)

S11 Fig An example to represent number of insertions and deletions in one cluster on *japonica* rice. Example of number of insertions and deletions present on cluster at 0.7 Mb having 8 genes on *japonica* rice chromosome 1. Horizontal lines indicated R-genes. Downward arrow of red colour indicated deletion and upward arrow of green colour indicated insertion. Scale is shown at bottom in basepairs.
(TIF)

S12 Fig Phylogenetic analysis of R-genes and DR-genes in *japonica* rice chromosomes. Phylogenetic analysis of R-genes and DR-genes present in clusters of 6 or more than 6 genes over 12 *japonica* rice chromosomes.
(TIF)

S13 Fig Synonymous and non-synonymous substitution of R-genes and DR-genes in clusters for *japonica* rice chromosome. Analysis of synonymous and non-synonymous substitution of R-genes and DR-genes in clusters of 6 or more than 6 genes over 12 *japonica* rice chromosomes depicting number of genes for a particular Ka/Ks range.
(TIF)

S14 Fig Phylogenetic analysis of DR-genes present on 12 *japonica* rice chromosomes. (TIF)

S15 Fig Analysis of synonymous and non-synonymous substitution of DR-genes of *japonica*. **(A)** Ka/Ks values for classes of DR-genes. **(B)** Number of DR-genes for a particular Ka/Ks range.
(TIF)

S1 Table Position, orientation and annotation of disease resistance and defense response genes in three rice genomes. (XLS)

S2 Table Distribution of clusters of R- & DR-genes on short and long arm of rice chromosomes. (DOC)

S3 Table Details of genes, exons, position, insertion and deletion in each cluster of R-genes and DR-genes. (DOC)

S4 Table Number of insertions and deletion in each cluster of R-genes & DR-genes over rice chromosomes. (DOC)

S5 Table Consensus sequences for the MEME defined motifs. (DOC)

S6 Table Number of R-& DR-genes having paralogs and their details in rice genome. (XLS)
