# Supplementary figures and images for: Genome-Wide Distribution, Organisation and Functional Characterization of Disease Resistance and Defence Response Genes across Rice Species
Source: PLoS One. 2015 Apr 22;10(4):e0125964. doi: 10.1371/journal.pone.0125964 (PMC4406684; doi:10.1371/journal.pone.0125964)

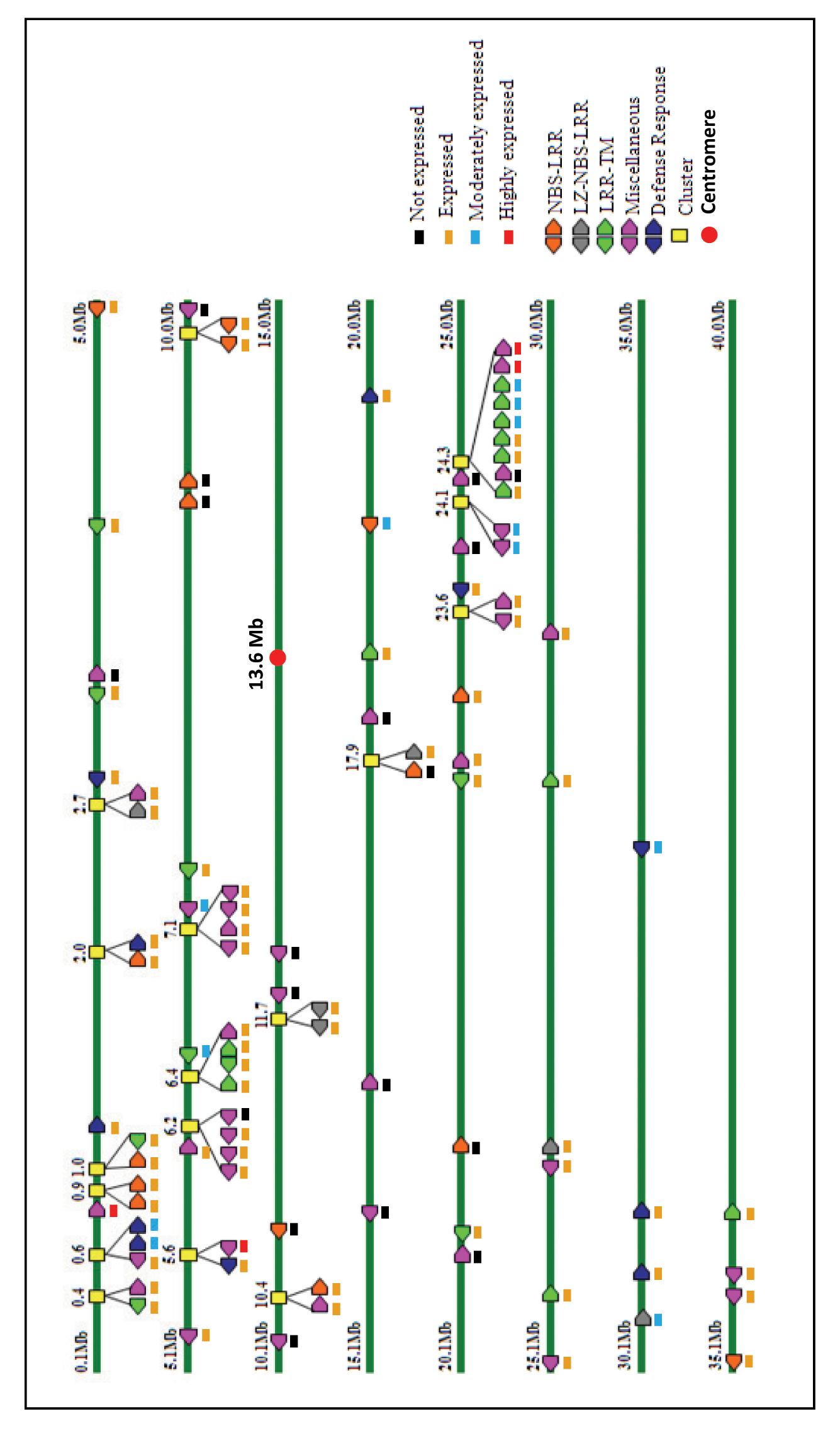

Supplement: S1 Fig — Example of physical position, orientation and expression of R-genes and DR-genes on japonica rice chromosome 2. Arrow heads of genes indicate their orientation. Rectangles against each gene showed their expression level based on EST matches. Position of each cluster in terms of Mb is given on the top of each line representing chromosomal segments. Class miscellaneous in figure stand for LRR (Leucine Rich Repeat). (TIF) [file pone.0125964.s001.tif]

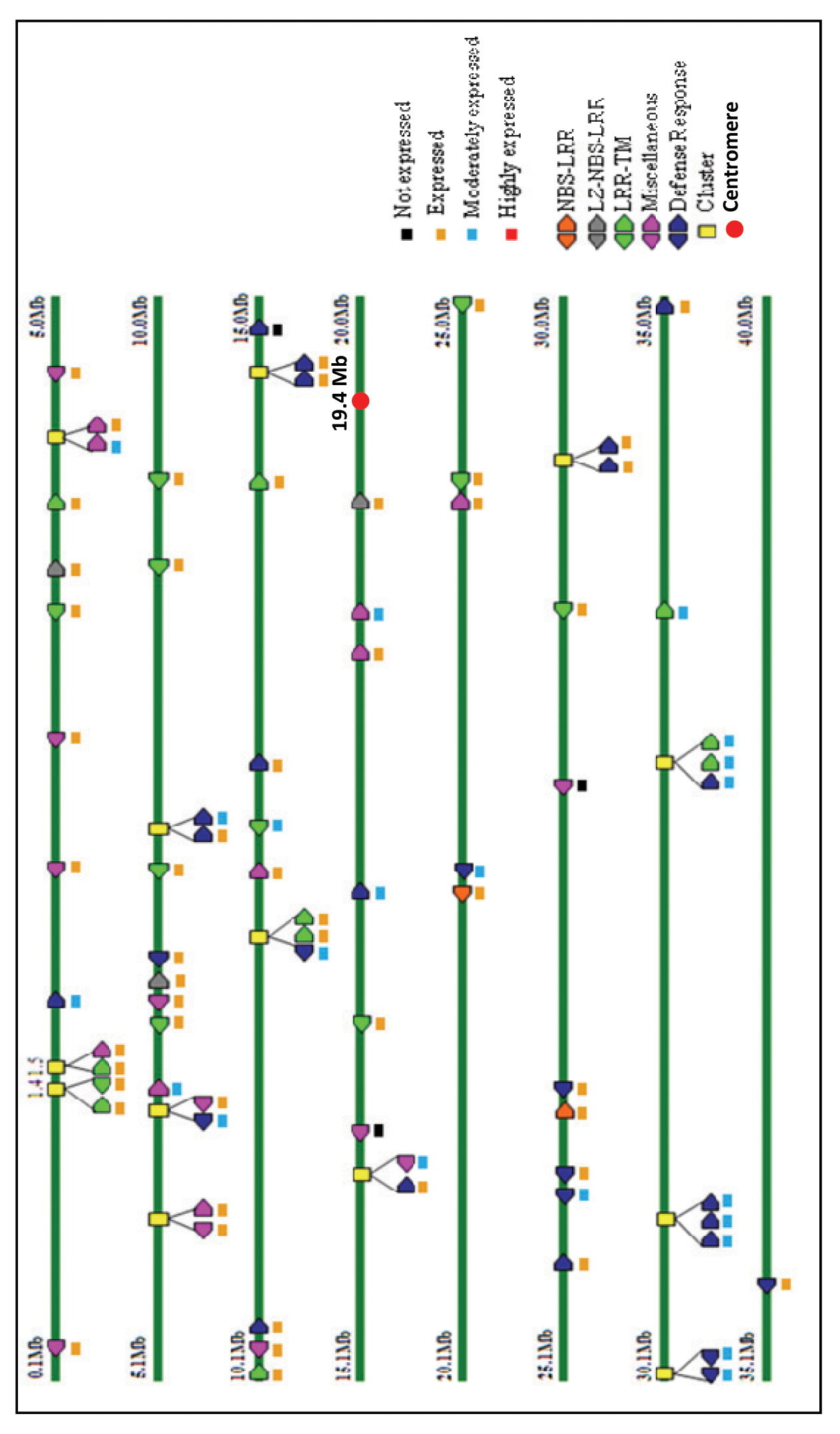

Supplement: S2 Fig — Example of physical position, orientation and expression of R-genes and DR-genes on japonica rice chromosome 3. Arrow heads of genes indicate their orientation. Rectangles against each gene showed their expression level based on EST matches. Position of each cluster in terms of Mb is given on the top of each line representing chromosomal segments. Class miscellaneous in figure stand for LRR (Leucine Rich Repeat). (TIF) [file pone.0125964.s002.tif]

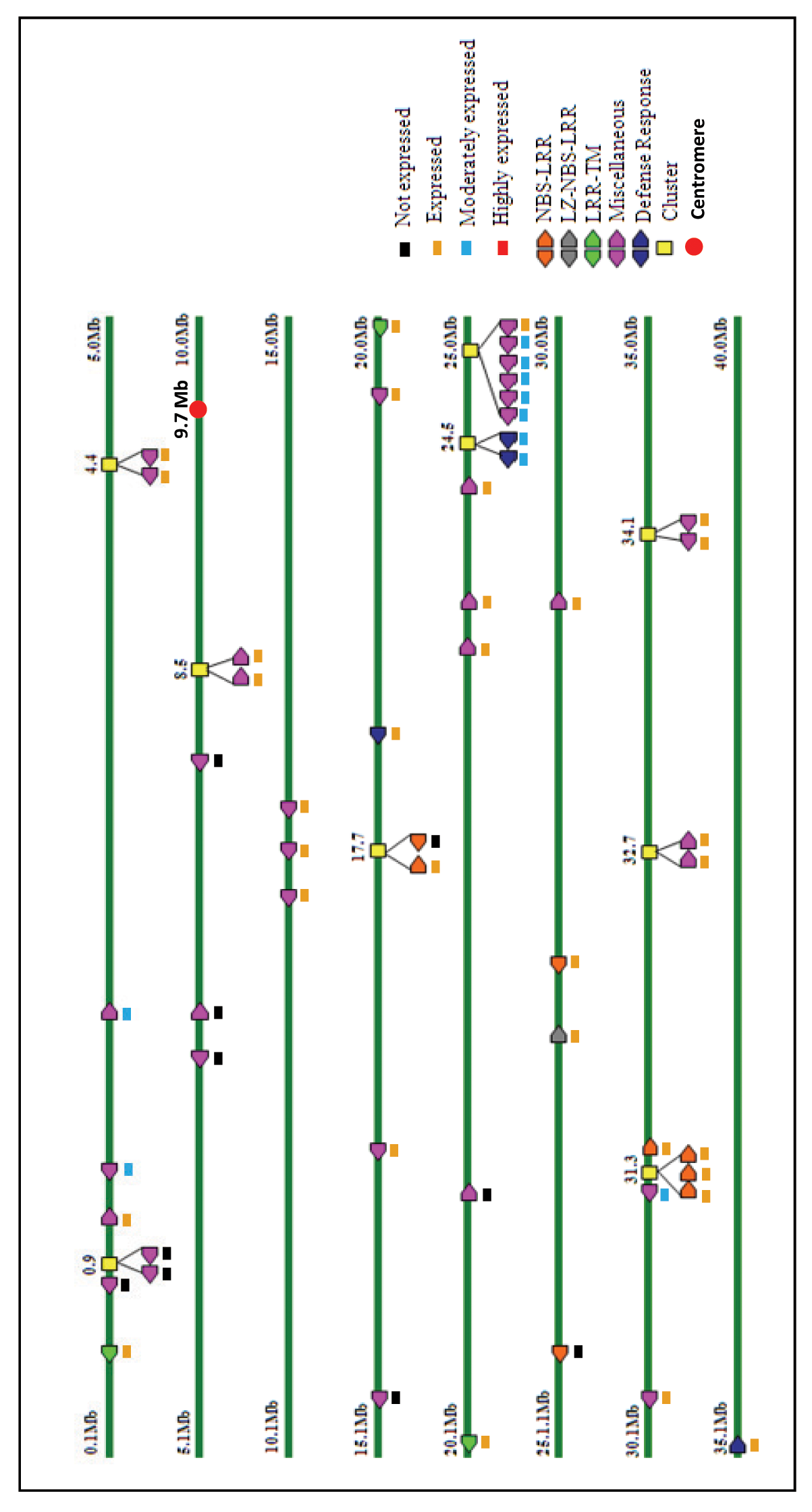

Supplement: S3 Fig — Example of physical position, orientation and expression of R-genes and DR-genes on japonica rice chromosome 4. Arrow heads of genes indicate their orientation. Rectangles against each gene showed their expression level based on EST matches. Position of each cluster in terms of Mb is given on the top of each line representing chromosomal segments. Class miscellaneous in figure stand for LRR (Leucine Rich Repeat). (TIF) [file pone.0125964.s003.tif]

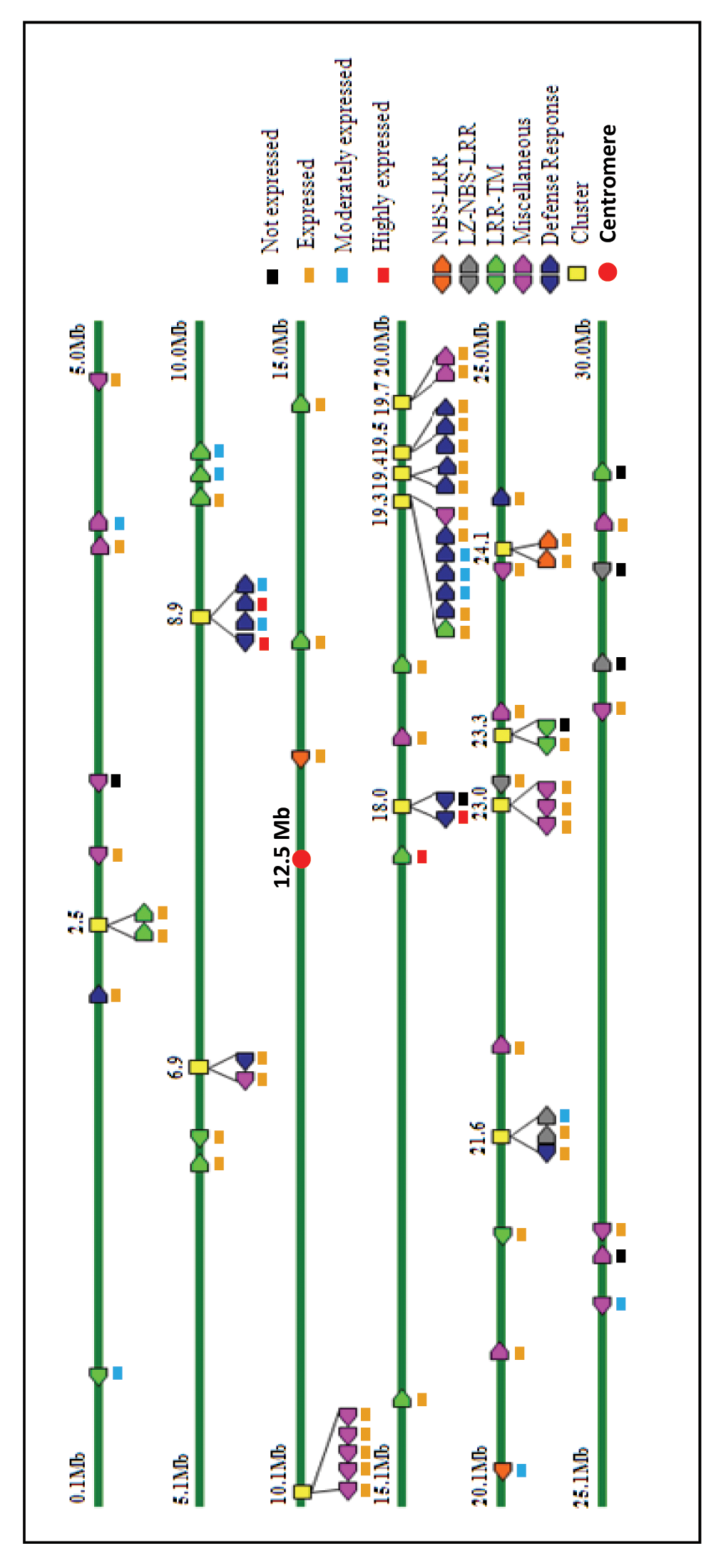

Supplement: S4 Fig — Example of physical position, orientation and expression of R-genes and DR-genes on japonica rice chromosome 5. Arrow heads of genes indicate their orientation. Rectangles against each gene showed their expression level based on EST matches. Position of each cluster in terms of Mb is given on the top of each line representing chromosomal segments. Class miscellaneous in figure stand for LRR (Leucine Rich Repeat). (TIF) [file pone.0125964.s004.tif]

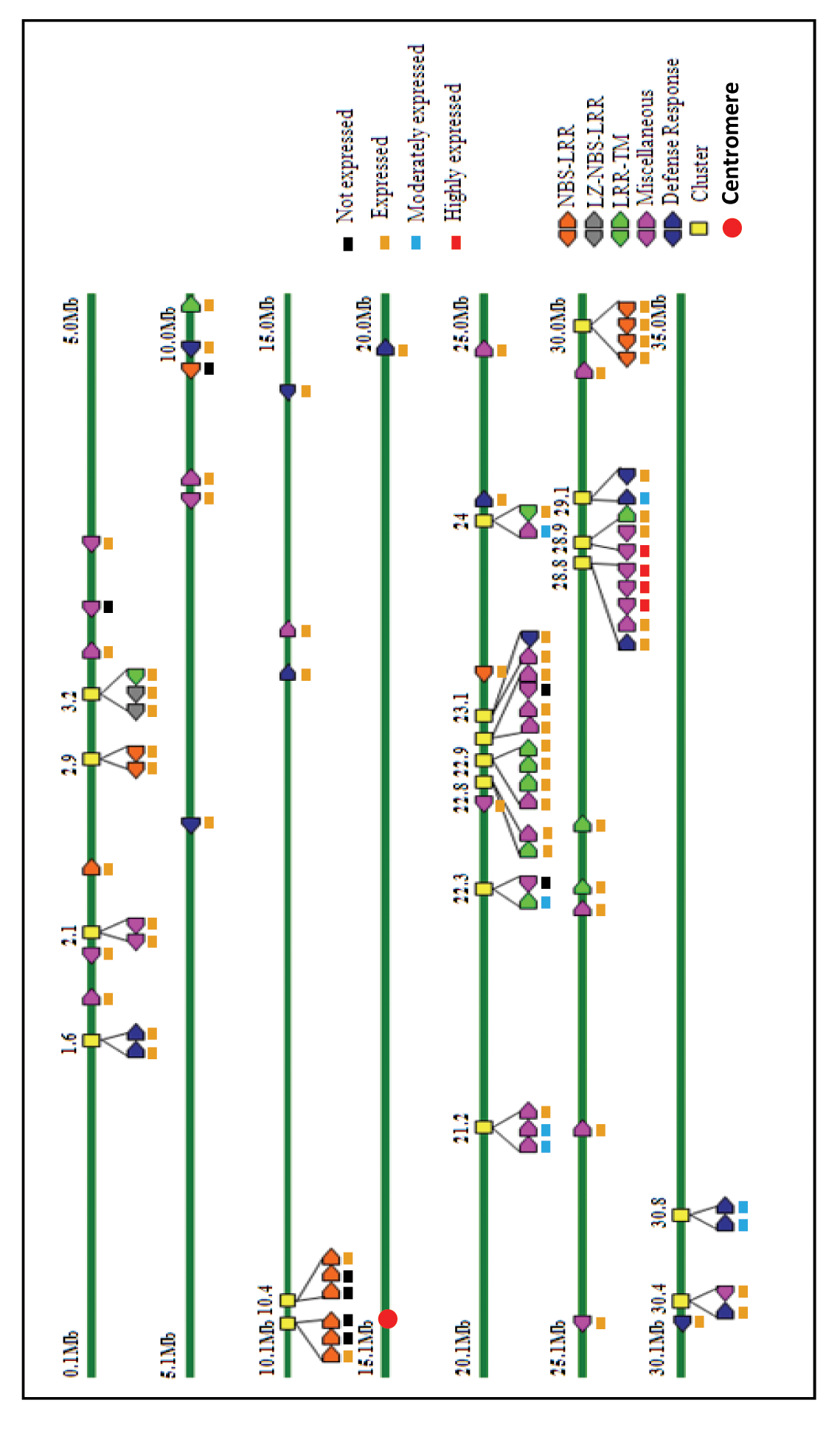

Supplement: S5 Fig — Example of physical position, orientation and expression of R-genes and DR-genes on japonica rice chromosome 6. Arrow heads of genes indicate their orientation. Rectangles against each gene showed their expression level based on EST matches. Position of each cluster in terms of Mb is given on the top of each line representing chromosomal segments. Class miscellaneous in figure stand for LRR (Leucine Rich Repeat). (TIF) [file pone.0125964.s005.tif]

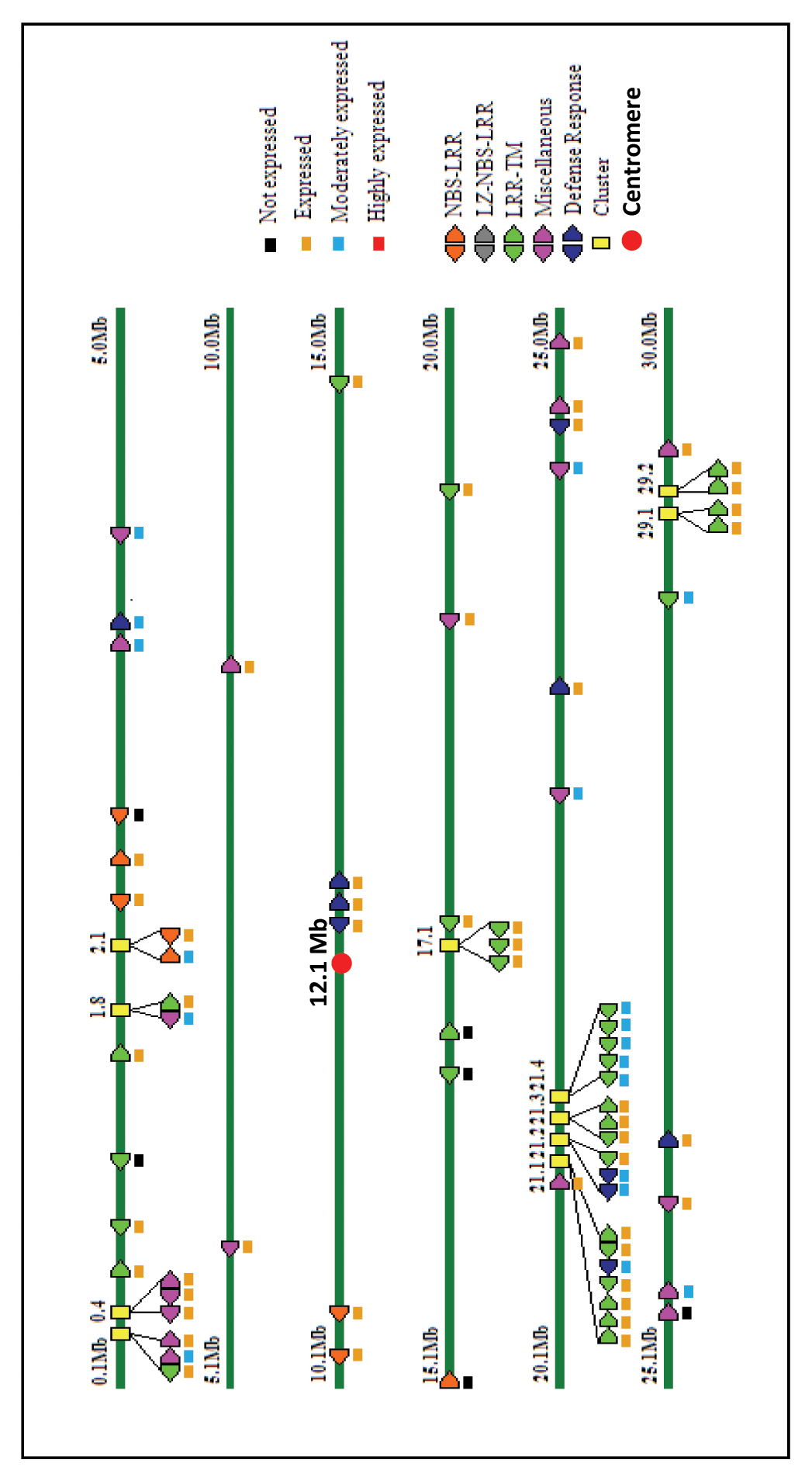

Supplement: S6 Fig — Example of physical position, orientation and expression of R-genes and DR-genes on japonica rice chromosome 7. Arrow heads of genes indicate their orientation. Rectangles against each gene showed their expression level based on EST matches. Position of each cluster in terms of Mb is given on the top of each line representing chromosomal segments. Class miscellaneous in figure stand for LRR (Leucine Rich Repeat). (TIF) [file pone.0125964.s006.tif]

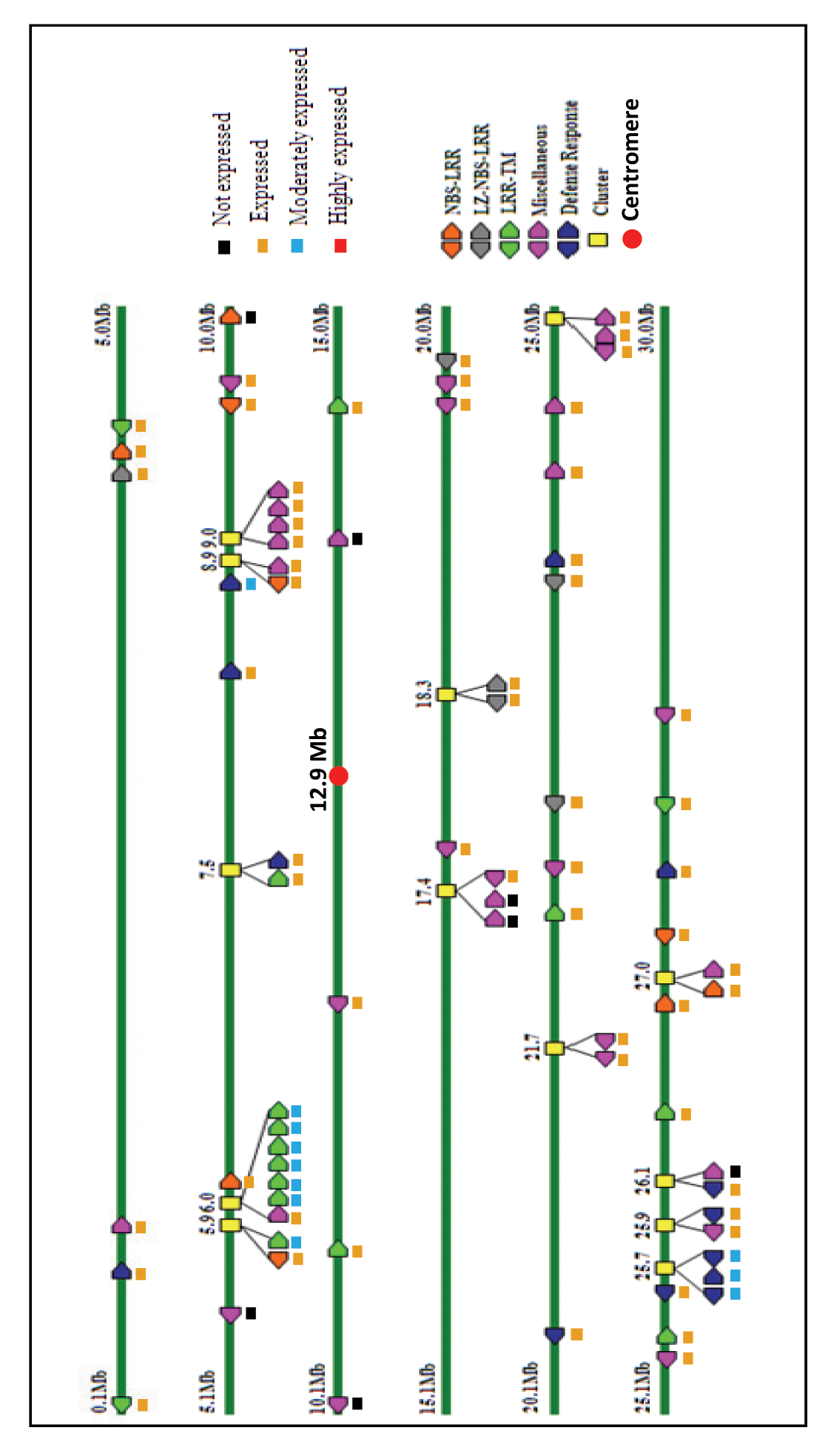

Supplement: S7 Fig — Example of physical position, orientation and expression of R-genes and DR-genes on japonica rice chromosome 8. Arrow heads of genes indicate their orientation. Rectangles against each gene showed their expression level based on EST matches. Position of each cluster in terms of Mb is given on the top of each line representing chromosomal segments. Class miscellaneous in figure stand for LRR (Leucine Rich Repeat). (TIF) [file pone.0125964.s007.tif]

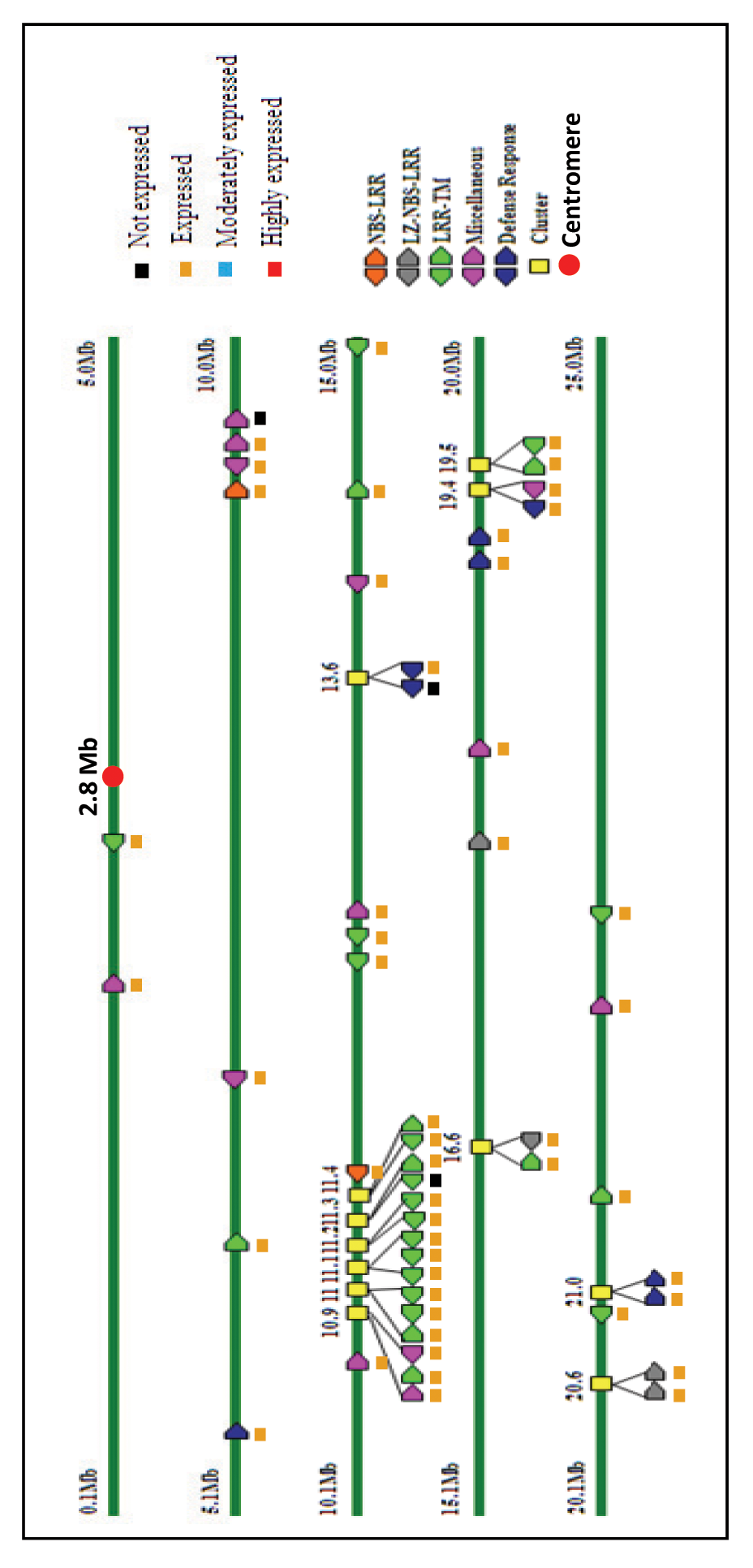

Supplement: S8 Fig — Example of physical position, orientation and expression of R-genes and DR-genes on japonica rice chromosome 9. Arrow heads of genes indicate their orientation. Rectangles against each gene showed their expression level based on EST matches. Position of each cluster in terms of Mb is given on the top of each line representing chromosomal segments. Class miscellaneous in figure stand for LRR (Leucine Rich Repeat). (TIF) [file pone.0125964.s008.tif]

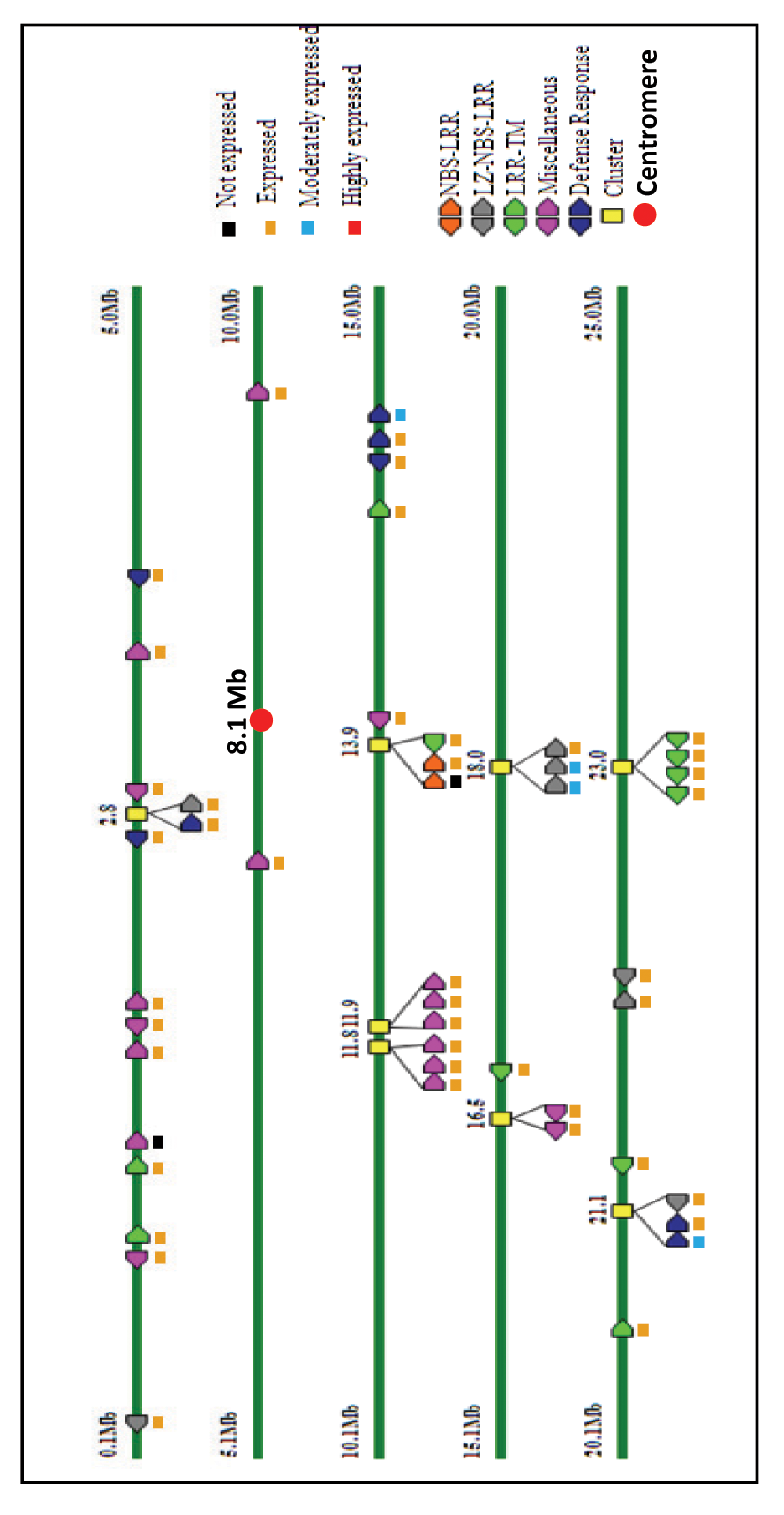

Supplement: S9 Fig — Example of physical position, orientation and expression of R-genes and DR-genes on japonica rice chromosome 10. Arrow heads of genes indicate their orientation. Rectangles against each gene showed their expression level based on EST matches. Position of each cluster in terms of Mb is given on the top of each line representing chromosomal segments. Class miscellaneous in figure stand for LRR (Leucine Rich Repeat). (TIF) [file pone.0125964.s009.tif]

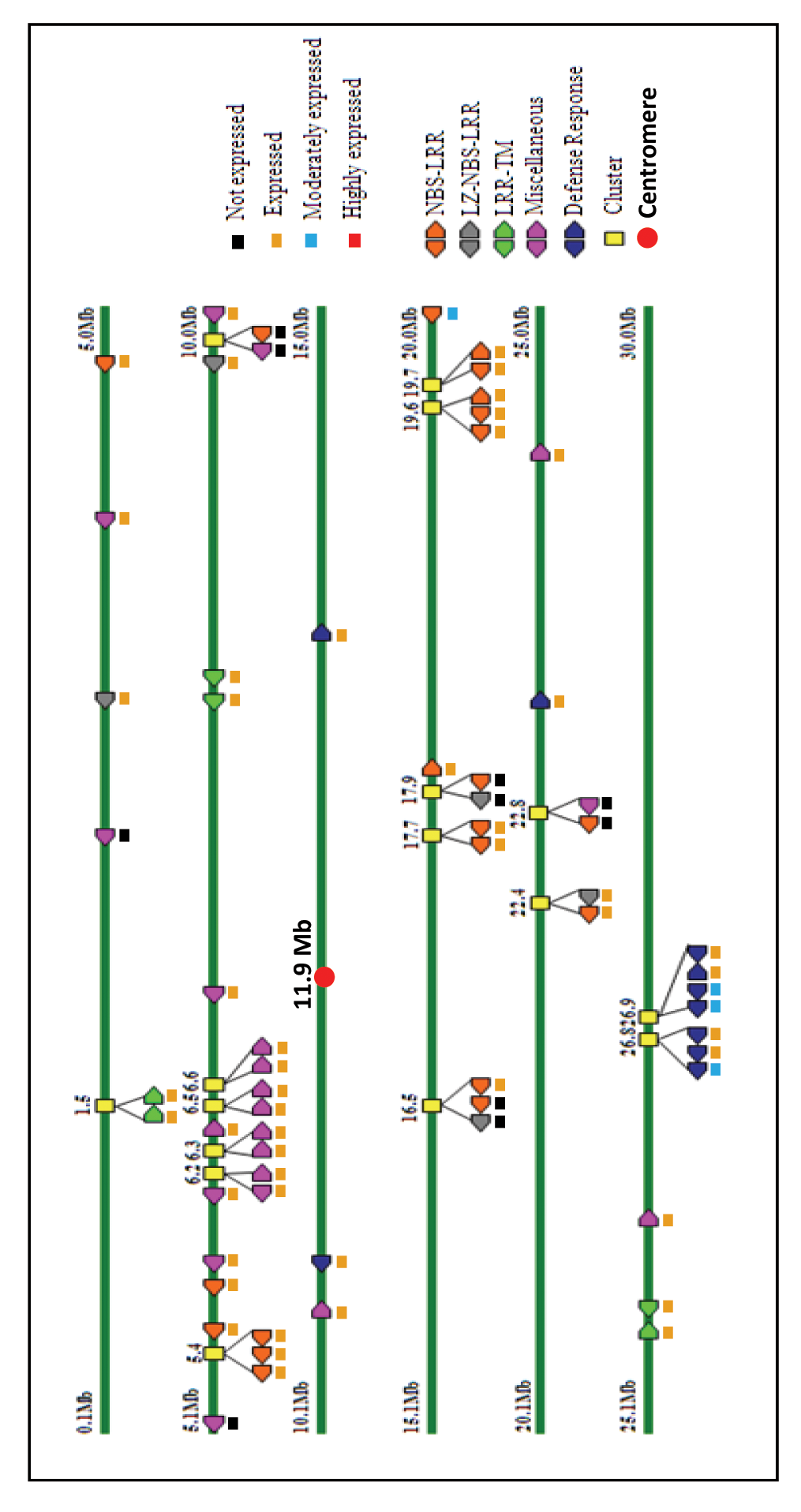

Supplement: S10 Fig — Example of physical position, orientation and expression of R-genes and DR-genes on japonica rice chromosome 12. Arrow heads of genes indicate their orientation. Rectangles against each gene showed their expression level based on EST matches. Position of each cluster in terms of Mb is given on the top of each line representing chromosomal segments. Class miscellaneous in figure stand for LRR (Leucine Rich Repeat). (TIF) [file pone.0125964.s010.tif]

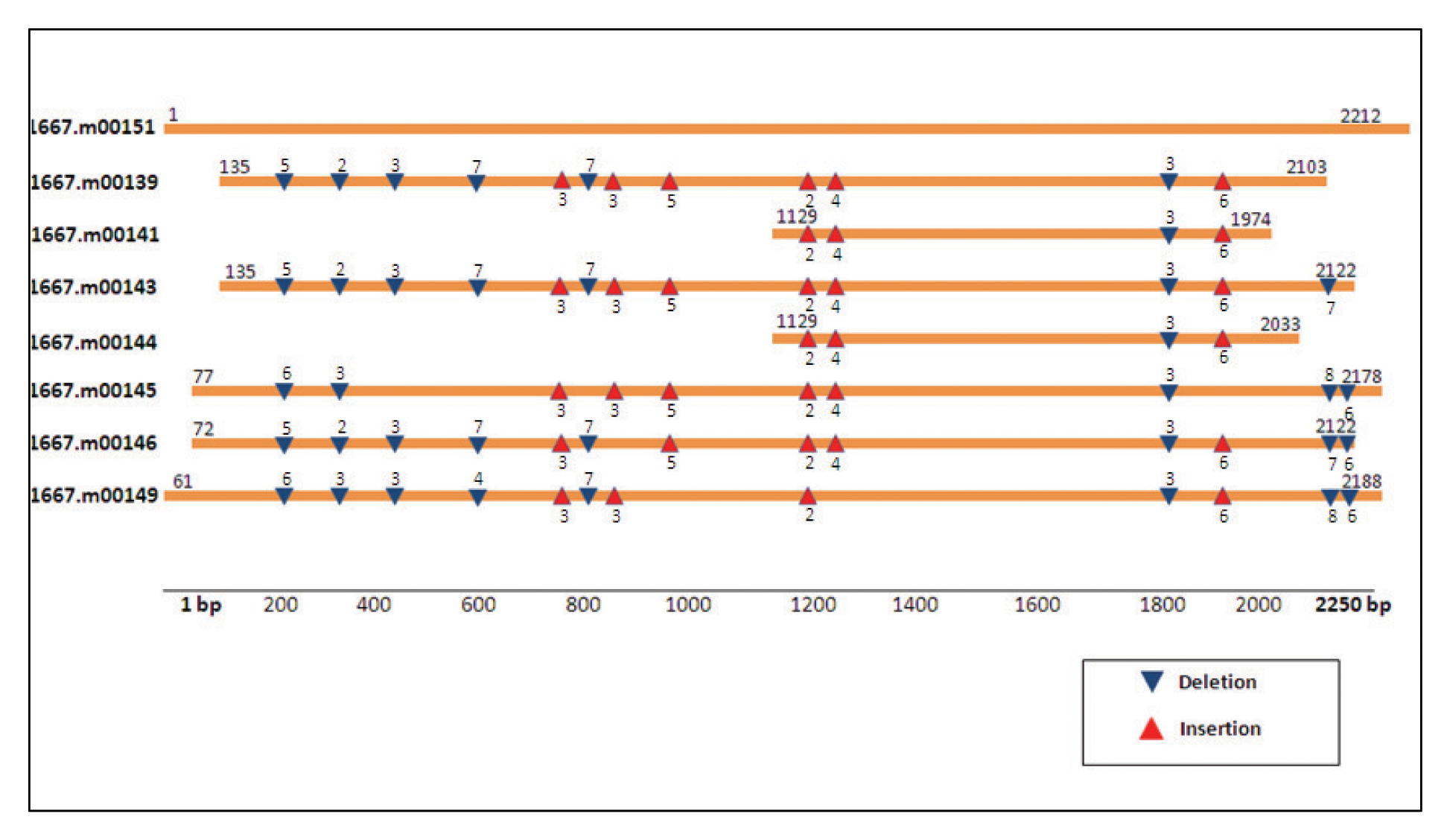

Supplement: S11 Fig — Example of number of insertions and deletions present on cluster at 0.7 Mb having 8 genes on japonica rice chromosome 1. Horizontal lines indicated R-genes. Downward arrow of red colour indicated deletion and upward arrow of green colour indicated insertion. Scale is shown at bottom in basepairs. (TIF) [file pone.0125964.s011.tif]

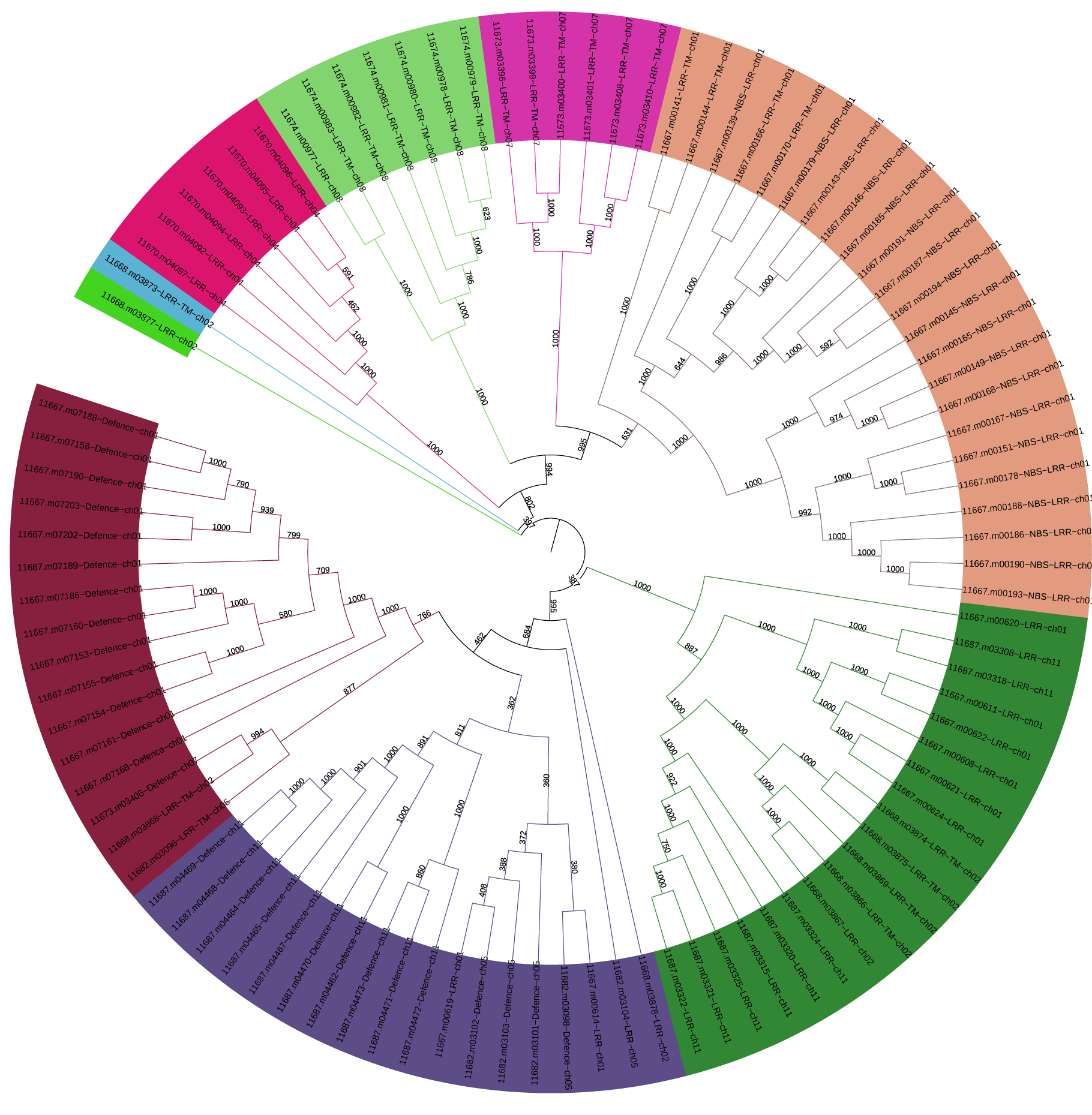

Supplement: S12 Fig — Phylogenetic analysis of R-genes and DR-genes present in clusters of 6 or more than 6 genes over 12 japonica rice chromosomes. (TIF) [file pone.0125964.s012.tif]

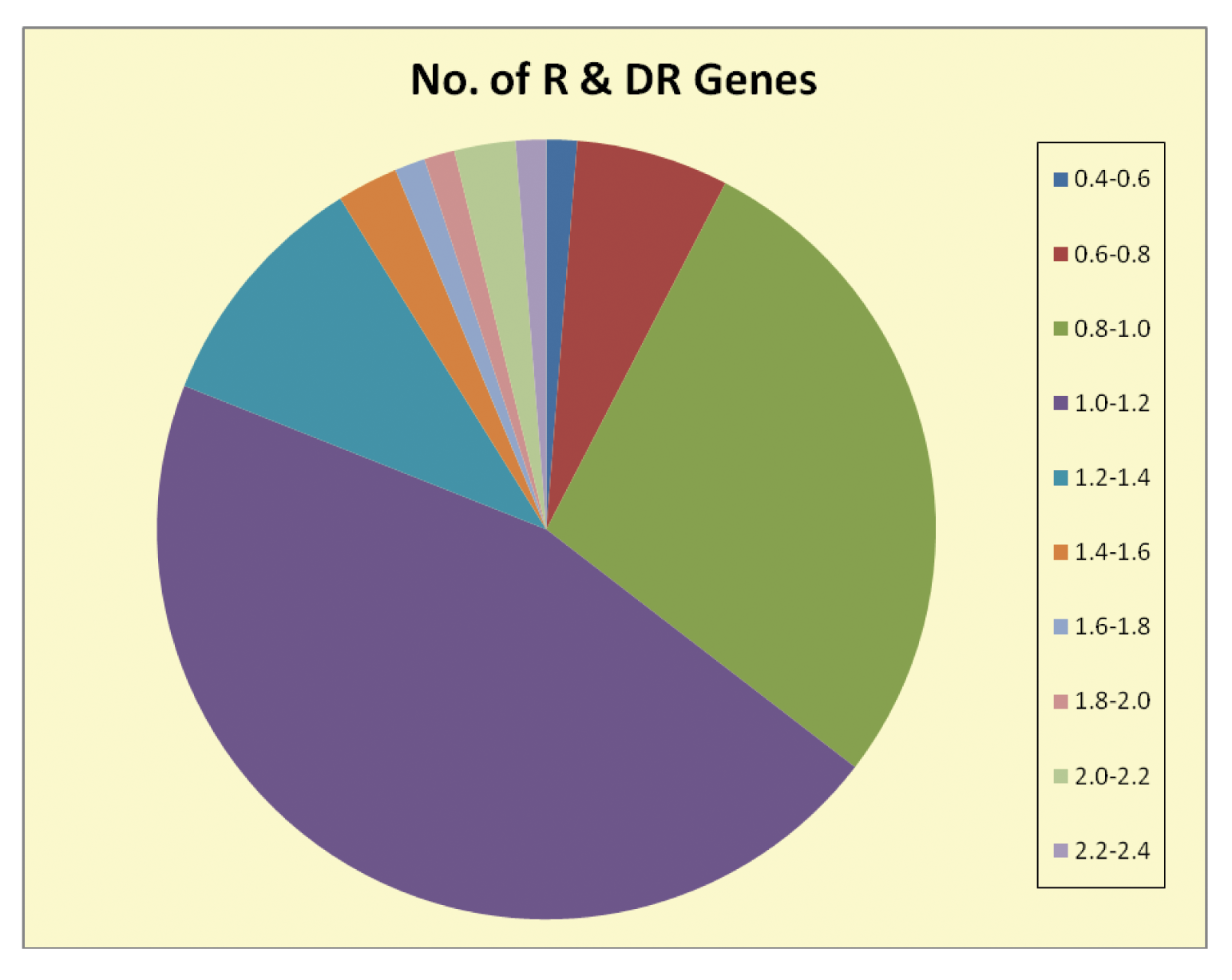

Supplement: S13 Fig — Analysis of synonymous and non-synonymous substitution of R-genes and DR-genes in clusters of 6 or more than 6 genes over 12 japonica rice chromosomes depicting number of genes for a particular Ka/Ks range. (TIF) [file pone.0125964.s013.tif]

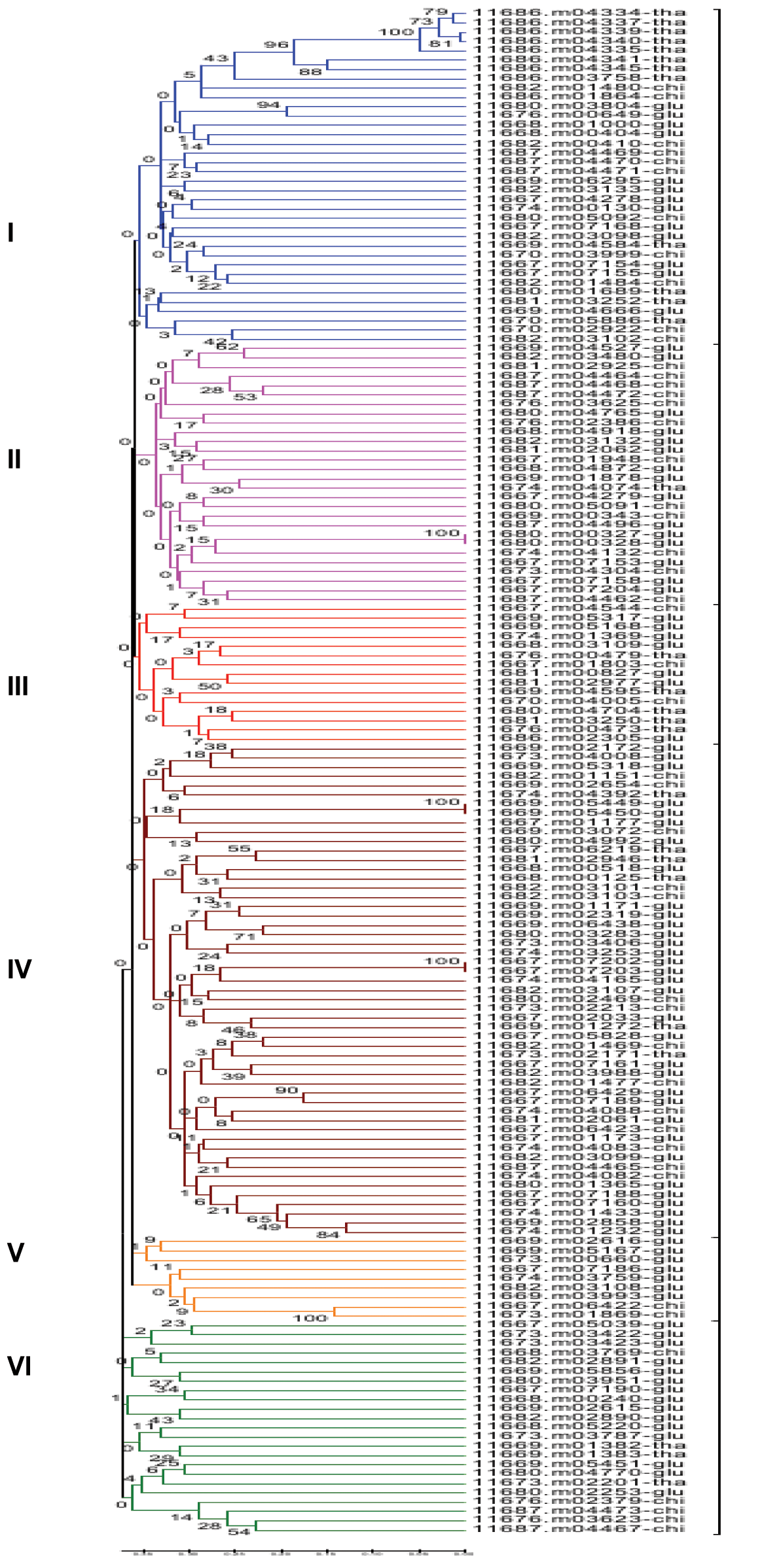

Supplement: S14 Fig — (TIF) [file pone.0125964.s014.tif]

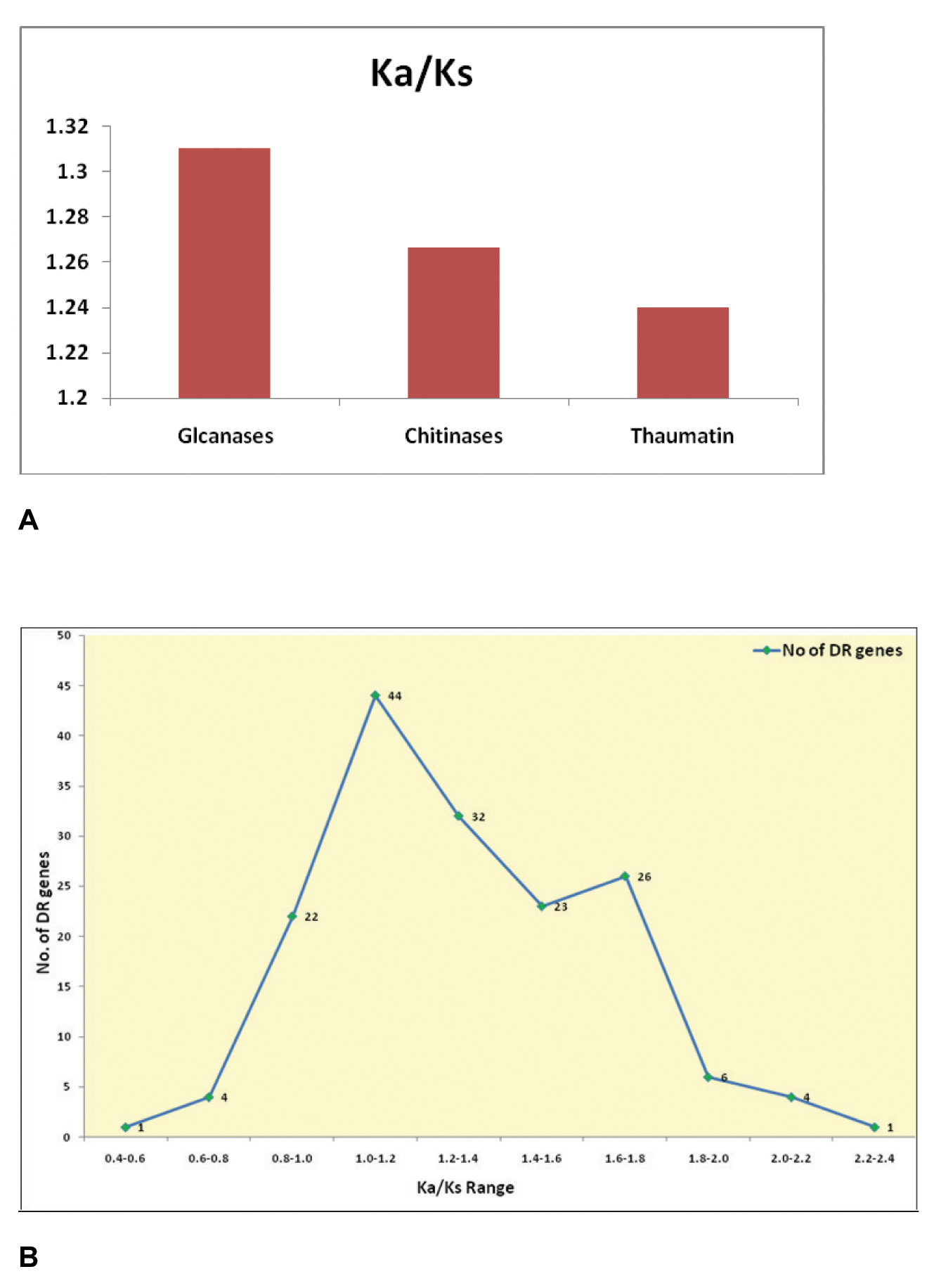

Supplement: S15 Fig — (A) Ka/Ks values for classes of DR-genes. (B) Number of DR-genes for a particular Ka/Ks range. (TIF) [file pone.0125964.s015.tif]
