# Supplementary material for: Genome-Wide Distribution, Organisation and Functional Characterization of Disease Resistance and Defence Response Genes across Rice Species
Source: PLoS One. 2015 Apr 22;10(4):e0125964. doi: 10.1371/journal.pone.0125964 (PMC4406684; doi:10.1371/journal.pone.0125964)
Supplement: S2 Table — (DOC) [file pone.0125964.s017.doc]

**S2 Table:** Distribution of clusters of R-genes & DR-genes and number of genes per cluster present on short arm and long arm of 12 rice chromosomes.

| **S.No.** | **Chr No.** | **No. of cluster** | | **Genes/cluster** | |
| --- | --- | --- | --- | --- | --- |
| 1.  2.  3.  4.  5.  6.  7.  8.  9.  10.  11.  12.  13. | 1  1  1  1  1  1  2  4  5  7  8  11  11 | **Short arm** | **Long arm** | **Short arm** | **Long arm** |
|  |  | 1  1  1  1  1 | 1  1  1  1  1  1  1  1 | 8  6  10  8  7 | 7  6  9  6  7  7  8  10 |
| **Total** |  | **5** | **8** | **39** | **60** |
