# Supplementary material for: Genome-Wide Distribution, Organisation and Functional Characterization of Disease Resistance and Defence Response Genes across Rice Species
Source: PLoS One. 2015 Apr 22;10(4):e0125964. doi: 10.1371/journal.pone.0125964 (PMC4406684; doi:10.1371/journal.pone.0125964)
Supplement: S3 Table — (DOC) [file pone.0125964.s018.doc]

**S3 Table:** No of exons in each gene, their position in bp, number of insertions and deletions in each cluster of 6 or more than 6 R-genes and DR-genes.

| **Clusters** | **Gene id** | **Exons**  **No.**  **(position, bp)** | **Insertions**  **No.**  **(position, no. of bp)** | **Deletions**  **No.**  **(position, no. of bp)** |
| --- | --- | --- | --- | --- |
| 1 | 11667.m00139 | 1 (61-1857) | 6  [768 (3), 930 (3), 990 (5), 1199 (2), 1216 (4), 1899 (6)] | 8  [213 (6) , 322 (2), 417 (3), 556 (7), 827 (7), 1837 (3), 2106 (8), 2126 (6)] |
|  | 11667.m00141 | 2 (1-706, 1242-1294) |  |  |
|  | 11667.m00143 | 1 (1-1848) |  |  |
|  | 11667.m00144 | 2 (1-768, 806-865) |  |  |
|  | 11667.m00145 | 1 (47-2037) |  |  |
|  | 11667.m00146 | 1 (131-2023) |  |  |
|  | 11667.m00149 | 1 (47-1980) |  |  |
|  | 11667.m00151 | 1 (1-2136) |  |  |
| 2 | 11667.m00165 | 1 (1-2202) | 13  [ 254 (3), 477(3), 520(3), 562(3), 1025(37), 1122(36), 1165(5), 1190, 1207, 1241, 1296, 1306, 1345] | 6  [28 (3), 118 (10), 698 (3), 1432 (3), 1975 (3), 2071(3)] |
|  | 11667.m00166 | 1 (1-1746) |  |  |
|  | 11667.m00167 | 1 (1-2019) |  |  |
|  | 11667.m00168 | 1 (1-1941) |  |  |
|  | 11667.m00170 | 1 (1-1803) |  |  |
|  | 11667.m00171 | No reliable prediction |  |  |
| 3 | 11667.m00178 | 1 (1-2010) | 16  [251 (6), 679 (3), 841(9), 1058 (6), 1081 (2), 1851 (5), 1877 (2), 1896 (5), 1935 (3),1947 (2), 1962 (5), 1974 (10), 2021 (2), 2030 (4), 2057 (4), 2101 (22)] | 1  [744 (6)] |
|  | 11667.m00179 | 1 (1-1926) |  |  |
|  | 11667.m00185 | 1 (1-1212) |  |  |
|  | 11667.m00186 | 1 (1-2139) |  |  |
|  | 11667.m00187 | 1 (183-2174) |  |  |
|  | 11667.m00188 | 1 (114-2194) |  |  |
|  | 11667.m00190 | 1 (1-2058) |  |  |
|  | 11667.m00191 | 1 (1-1926) |  |  |
|  | 11667.m00193- | 1 (1-2121) |  |  |
|  | 11667.m00194 | 1 (1-1911) |  |  |
| 4 | 11667.m00608 | 1 (1-2203) | 1  [3155 (1)] | 4  [1177 (3), 1252 (8), 1341 (5), 1866 (3)] |
|  | 11667.m00611 | 1(1-2982) |  |  |
|  | 11667.m00614 | 1 (1-1005) |  |  |
|  | 11667.m00619 | 1 (1-1471) |  |  |
|  | 11667.m00620 | 1 (1-2544) |  |  |
|  | 11667.m00621 | 1 (1-2289) |  |  |
|  | 11667.m00622 | 1 (1-3093) |  |  |
|  | 11667.m00624 | 1 (197-3187) |  |  |
| 5 | 11667.m07153 | 1 (102 – 1094) | 6  [192 (2), 306 (4), 1180 (65), 2160 (85), 2367 (130), 2706 (1200)] | 1  [142 (2)] |
|  | 11667.m07154 | 1 (29 – 1027) |  |  |
|  | 11667.m07155 | 1 (30 – 964) |  |  |
|  | 11667.m07158 | 1 (30 – 1034) |  |  |
|  | 11667.m07160 | 1 (1 – 1014) |  |  |
|  | 11667.m07161 | 1 (1 – 1092) |  |  |
|  | 11667.m07168 | 1 (80 – 1000) |  |  |
| 6 | 11667.m07186 | 1 (1 – 693) | 1  [782 (1)] | 5  [183 (2), 433 (1), 519 (2), 738 (1), 761 (1)] |
|  | 11667.m07188 | 1 (1 – 1005) |  |  |
|  | 11667.m07189 | 1 (80 – 1014) |  |  |
|  | 11667.m07190 | 1 (1363 – 2287) |  |  |
|  | 11667.m07202 | 1 (1 – 957) |  |  |
|  | 11667.m07203 | 1 (1 – 957) |  |  |
| 7 | 11668.m03866 | 1 (1 – 2994) | 5  [264 (9), 1064 (7), 1688 (14), 1864 (6), 2300 (10)] | 5  [740 (48), 861 (54), 932 (8), 2249 (3), 2750 (3)] |
|  | 11668.m03867 | 1 (1 – 2994) |  |  |
|  | 11668.m03868 | 1 (256 – 918) |  |  |
|  | 11668.m03869 | 2 (185 – 3188, 3252 – 3319) |  |  |
|  | 11668.m03873 | 1 (1-3264) |  |  |
|  | 11668.m03874 | 1 (149 – 3154) |  |  |
|  | 11668.m03875 | 1 (77 – 3082) |  |  |
|  | 11668.m03877 | 1(1 – 3495) |  |  |
|  | 11668.m03878 | 1 (40 – 1356) |  |  |
| 8 | 11670.m04092 | 1 (205 – 1950) | 10  [344 (2), 497 (2), 515 (1), 808 (1), 828 (3), 867 (6), 1138 (9), 1156 (32), 1197 (23), 1231 (11)] | 3  [476 (3), 691 (9), 1319 (6)] |
|  | 11670.m04093 | 1 (312 – 2057) |  |  |
|  | 11670.m04094 | 1 (307 – 2055) |  |  |
|  | 11670.m04095 | 1 (307 – 2133) |  |  |
|  | 11670.m04096 | 1 (307 – 1923) |  |  |
|  | 11670.m04097 | 1 (205 – 1431) |  |  |
| 9 | 11682.m03096 | 1 (23 – 1522) | 5  [382 (1), 484 (2), 660 (3), 674 (2), 835 (2) | 0 |
|  | 11682.m03098 | 2 (215 – 470, 753 – 937) |  |  |
|  | 11682.m03099 |  |  |  |
|  | 11682.m03101 | 1 (13 – 1035) |  |  |
|  | 11682.m03102 | 1 (92 – 1048) |  |  |
|  | 11682.m03103 | 1 (1 – 1023) |  |  |
|  | 11682.m03104 | 1 (1-768) |  |  |
| 10 | 11673.m03396 | 2 (1 – 883, 957 – 964) | 8  [162 (1), 519 (3), 1584 (2), 1740 (4), 1751 (2), 1809 (5), 1827 (5), 1898 (8)] | 0 |
|  | 11673.m03399 | 2 (1-701, 1037-1040) |  |  |
|  | 11673.m03400 | 2 (1-735, 912-977) |  |  |
|  | 11673.m03401 | 1 (131 – 1080) |  |  |
|  | 11673.m03406 | 3 (61 – 756, 1075 – 1165, 1231 – 1253) |  |  |
|  | 11673.m03408 | 1 (258 – 2243) |  |  |
|  | 11673.m03410 | 1 (1 – 1917) |  |  |
| 11 | 11674.m00977 | 1 (1-3072) | 5  [169 (4), 251 (2), 340 (6), 412 (3), 823 (7)] | 4  [657 (9), 833 (5), 1067 (5), 1085 (4)] |
|  | 11674.m00978 | 3 (74 -2075, 2576 – 2620, 2749 – 3491) |  |  |
|  | 11674.m00979 | 2 (74 – 2497, 2550 – 3122) |  |  |
|  | 11674.m00980 | 1 (74 – 3148) |  |  |
|  | 11674.m00981 | 1 (14 – 3061) |  |  |
|  | 11674.m00982 | 4 (24 – 167, 445 – 516, 753 – 965, 1033 – 3001) |  |  |
|  | 11674.m00983 | 1 (77 – 3148) |  |  |
| 12 | 11687.m03308 | 1 (1 – 1938) | 5  [122 (3), 357 (3), 432 (3), 630 (8), 750 (3)] | 2  [739 (3), 771 (3)] |
|  | 11687.m03315 | 1 (1 – 3402) |  |  |
|  | 11687.m03318 | 1 (1 – 2268) |  |  |
|  | 11687.m03320 | 1 (1 – 3207) |  |  |
|  | 11687.m03321 | 1 (1 – 3108) |  |  |
|  | 11687.m03322 | 1 (1 – 3108) |  |  |
|  | 11687.m03324 | 1 (1 – 3114) |  |  |
|  | 11687.m03325 | 1 (88 – 2376) |  |  |
| 13 | 11687.m04462 | 1 (36 – 950) | 1  [1931 (6)] | 4  [1664 (3), 1739 (3), 2077 (9), 1970 (3) |
|  | 11687.m04464 | 1 (58 – 930) |  |  |
|  | 11687.m04465 | 1 (115 – 993) |  |  |
|  | 11687.m04467 | 1 (80 – 949) |  |  |
|  | 11687.m04468 | 1 (40 – 894) |  |  |
|  | 11687.m04469 | 1 (15 – 854) |  |  |
|  | 11687.m04470 | 1 (108 – 1022) |  |  |
|  | 11687.m04471 | 1 (78 – 980) |  |  |
|  | 11687.m04472 | 1 (37 – 942) |  |  |
|  | 11687.m04473 | 1 (62 – 970) |  |  |
