# Supplementary material for: Genome-Wide Distribution, Organisation and Functional Characterization of Disease Resistance and Defence Response Genes across Rice Species
Source: PLoS One. 2015 Apr 22;10(4):e0125964. doi: 10.1371/journal.pone.0125964 (PMC4406684; doi:10.1371/journal.pone.0125964)
Supplement: S4 Table — (DOC) [file pone.0125964.s019.doc]

**S4 Table:** Number of insertions and deletion in each cluster of 6 or more than 6 R-genes & DR-genes over 12 rice chromosomes.

| **S. No.** | **Cluster** | **Insertion** | **Deletion** |
| --- | --- | --- | --- |
| 1 | cluster 1 | 6 | 6 |
| 2 | cluster 2 | 13 | 6 |
| 3 | cluster 3 | 16 | 1 |
| 4 | cluster 4 | 1 | 4 |
| 5 | cluster 5 | 6 | 1 |
| 6 | cluster 6 | 1 | 5 |
| 7 | cluster 7 | 5 | 5 |
| 8 | cluster 8 | 10 | 3 |
| 9 | cluster 9 | 5 | 0 |
| 10 | cluster 10 | 8 | 0 |
| 11 | cluster 11 | 5 | 4 |
| 12 | cluster 12 | 5 | 2 |
| 13 | cluster 13 | 1 | 4 |
| **Total** | **13** | **82** | **41** |
