# Supplementary material for: Genome-Wide Distribution, Organisation and Functional Characterization of Disease Resistance and Defence Response Genes across Rice Species
Source: PLoS One. 2015 Apr 22;10(4):e0125964. doi: 10.1371/journal.pone.0125964 (PMC4406684; doi:10.1371/journal.pone.0125964)
Supplement: S5 Table — (DOC) [file pone.0125964.s020.doc]

**S5 Table:** Consensus sequences for the MEME defined motifs.

| **S.No.** | **Motifs** | **Consensus sequence** |
| --- | --- | --- |
| **1** | Motif 1 | IPISLGNLKGLSVLNLSHNILSGTIPAVLGDLPLLSKLDLSYNNLQGEIP |
| **2** | Motif 2 | KAFANMVNLGSLDLERCPKIHGGLVHLKGLRKLEKLNLRYC |
| **3** | Motif 3 | NAFDGPIPSSLGNLSGLQKLYLSHNNLEG |
| **4** | Motif 4 | TAECEALRNLRHRNLVKIITACSSIDNSGNDFKAIVFDFMP |
| **5** | Motif 5 | QRVGILLDVANALDYLHCHGPTPVVHCDLKPSNVLLDAEMVAHVGDFGLA |
| **6** | Motif 6 | TSSMGLRGTIGYAPPEYGAGNTVSTQGDIYSYGILVLETVTGKRPTDKKF |
| **7** | Motif 7 | GNLTDLHTLDLSSNNFSGQIP |
| **8** | Motif 8 | ACLEVISGLASLVLLNLSRCGVYDEGCEHLEGLVKLKVLNLGFNYITDAC |
| **9** | Motif 9 | WHKKIQTEIPSTTSMRGHPLVSYSQLVKATDEFSIANLLGSGSFGSVYKG |
| **10** | Motif 10 | WNTSTHFCRWQGVKCTSTGPW |
| **11** | Motif 11 | YKVMIDCLVSLLRLGLYCSQEIPSNRMSTGDIIKELNAIKQ |
| **12** | Motif 12 | IFANPTVVSVQGNIGLCGGVMDLRMPPCQVVSQRRKTQYYL |
| **13** | Motif 13 | QISEHGLKTLSGLSNVTSLSFKKCSAVTA |
| **14** | Motif 14 | SIPQEIGHLKNLVEF |
| **15** | Motif 15 | SKLQTLDLGENNLGGVLPNSF |
| **16** | Motif 16 | GLSLREYVELGLHGKMMDVVDTQLFLGLE |
| **17** | Motif 17 | TTFKMIDLSNNDFNG |
| **18** | Motif 18 | VKIQVAIKVFDLEMRWADKSF |
| **19** | Motif 19 | VRCPSLMELCVAKVRE |
| **20** | Motif 20 | ALDRCQNLVTIQMDQNFLTGT |
